# Nanoplastics-mediated physiologic and genomic responses in pathogenic *Escherichia coli* O157:H7

**DOI:** 10.1186/s12951-025-03369-z

**Published:** 2025-04-21

**Authors:** Jayashree Nath, Goutam Banerjee, Jayita De, Noella Dsouza, Shantanu Sur, John W. Scott, Pratik Banerjee

**Affiliations:** 1 Department of Food Science and Human Nutrition, University of Illinois at Urbana-Champaign, Urbana, IL 61801 USA; 2 Department of Biology, Clarkson University, Potsdam, NY 13699 USA; 3 Prairie Research Institute, Illinois Sustainable Technology Center, University of Illinois, Champaign, IL 61820 USA

**Keywords:** Nanoplastics, Microplastics, *E. coli* O157:H7, Biofilms, Virulence, Global gene expression

## Abstract

The widespread occurrence of microplastics (MP) and nanoplastics (NP) in the environment is commonly thought to negatively impact living organisms; however, there remains a considerable lack of understanding regarding the actual risks associated with exposure. Microorganisms, including pathogenic bacteria, frequently interact with MPs/NPs in various ecosystems, triggering physiological responses that warrant a deeper understanding. The present study experimentally demonstrated the impact of surface-functionalized differentially charged polystyrene (PS) NPs on the physiology of human pathogenic *Escherichia coli* O157:H7 and their influence on biofilm formation. Our results suggest that charged NPs can influence the growth, viability, virulence, physiological stress response, and biofilm lifestyle of the pathogen. Positively-charged NPs were found to have a bacteriostatic effect on planktonic cell growth and affect cellular viability and biofilm initiation compared to negatively charged and uncharged NPs. The transcriptomic and gene expression data indicated significant changes in the global gene expression profile of cells exposed to NPs, including the differential expression of genes encoding several metabolic pathways associated with stress response and virulence. Significant upregulation of Shiga-like toxin (*stx*_*1a*_), quorum sensing, and biofilm initiation genes was observed in NP-exposed biofilm samples. Overall, exposure to NPs did not significantly affect the survival of pathogens but affected their growth and biofilm development pattern, and most importantly, their virulence traits.

**Graphical abstract:**

**Figure:**
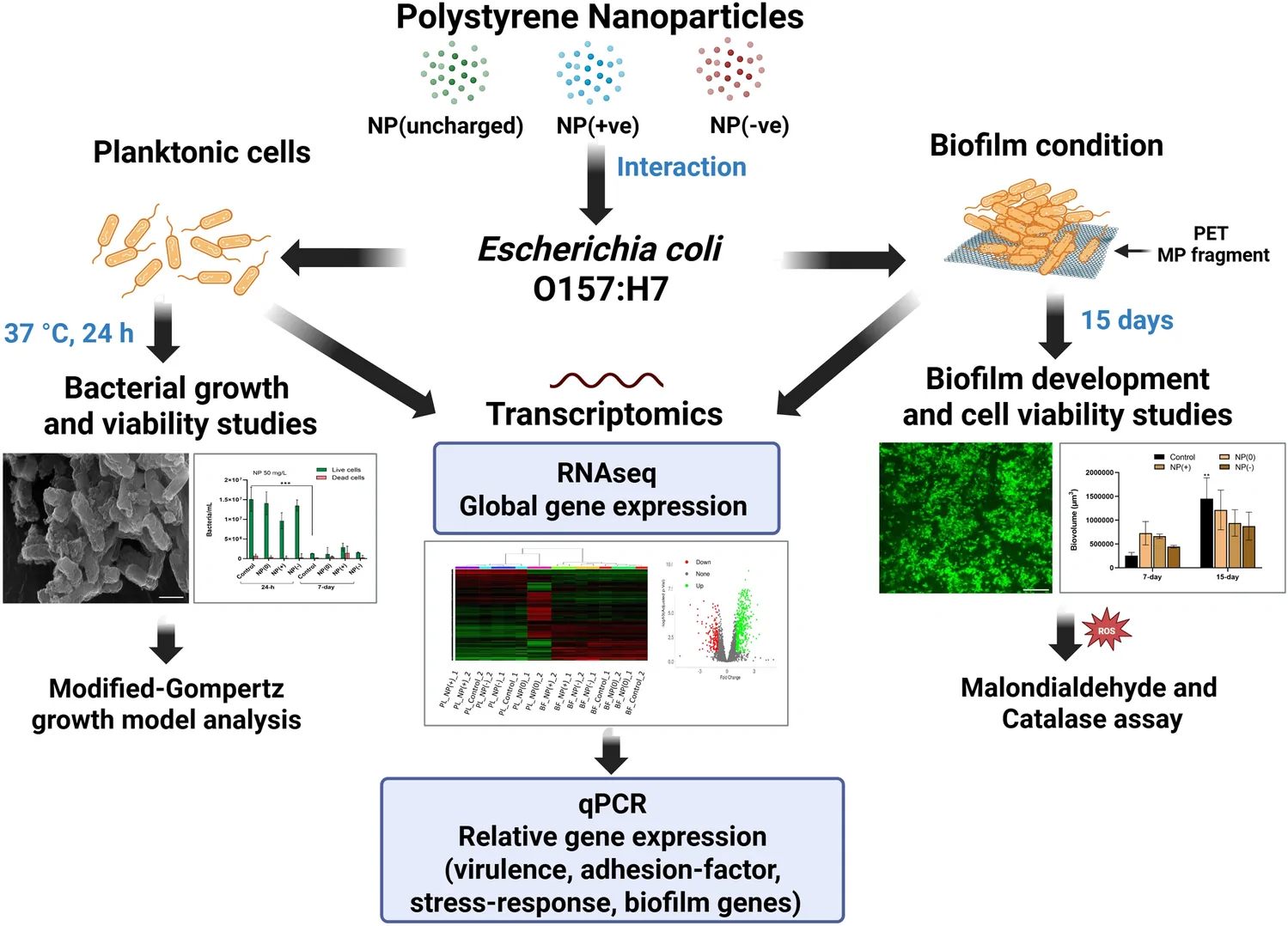

**Supplementary Information:**

The online version contains supplementary material available at https://doi.org/10.1186/s12951-025-03369-z.

## Introduction

The extensive use and improper disposal of plastic materials by humans poses a substantial threat to both the environment and human health. The impact of transmission and proliferation of plastics and plastic fragments—microplastics (MPs) size ≤ 5 mm and nanoplastics (NPs) size ≤ 100 nm, on human and animal health is not fully understood [1]. Recent studies have evaluated the interaction of surface charged NPs with bacterial membrane or cell envelop [2]; however, little is known about the impact of MPs/NPs on the physiology of human pathogenic bacteria [3]. It is intuitive to assume that in natural environments, persistent pathogenic microbes are exposed to plastics of varying sizes, morphologies, chemical compositions, and adsorbed chemical species, rendering surface charge to the plastics [4]. Smaller NPs have been reported to aggregate more on the bacterial cell surface under the same conditions as larger particles [5]. The size of the plastic particle is important for their interaction with microbes [6]. Studies on the interactions of bacteria with different sizes of MPs/NPs show that NPs, owing to their larger surface-area-to-size ratio, influence microbial physiology and activities through mechanisms very similar to those of metal-based nanoparticles [7, 8]. The binding of NPs to the bacterial cell surface leads to the onset of signaling cascades, although the exact mechanism or process is not fully understood [3, 6]. On the contrary, MPs larger than microbes in overall size provide a platform for selective attachment and biofilm formation, increasing the probability of microbial interactions with other environmental pollutants. NPs trigger global expression of microbial genes and pathways that can affect microbial functionality in the ecosystem [3, 9]. In real environments, the complex surface composition of NPs, due to the natural process of degradation, and sorption of other charged species is unavoidable [10–12], which increases the toxicity of NPs on soil microbiota, causing microbial community shifts [13].

The toxicological effects of NPs have been demonstrated in a variety of microbes, including *Escherichia coli* (non-pathogenic strains) [2, 14]*;* however*,* their impact on human pathogens remains less explored [3]. *E. coli* serotype O157:H7 is a Shiga toxin producing strain that disrupts protein synthesis of endothelial cells resulting to bloody diarrhea and has been linked with several outbreaks worldwide [15, 16]. The persistence of *E. coli* O157:H7 in the environment (water, soil, sludge, food-processing equipment, etc.) has been a perpetual concern [17, 18]. It should be noted that most studies exploring the interaction of NPs with bacteria were conducted using planktonic cells [3]; however, performing these studies on bacterial biofilms better emulates the real environment [15] where biofilms form [19]. Biofilms often serve as a hotspot for the exchange of genetic material, which may influence their virulence traits and, in turn, may enable pathogens with better survivability. This study experimentally demonstrated the altered growth pattern and expression of genes in the Gram-negative human pathogenic strain *E. coli* O157:H7 EDL 933, upon exposure to differentially surface charged NPs both in planktonic conditions and during biofilm formation. To achieve this aim, we exposed the Gram-negative human pathogenic strain *E. coli* O157:H7 EDL 933 to three categories of differentially charged polystyrene-based NPs: carboxyl-modified, negatively charged NP(−), amine-modified, positively charged NP( +), and uncharged NP(0), with a similar size range (30–100 nm). We evaluated the growth pattern, biofilm formation, and changes in the expression of virulence factors, adhesion factors, stress responses, and biofilm-related genes in planktonic *E. coli* O157: H7 cells and biofilms grown on PET-based microplastic (MP) fragments. The impact of these differentially charged NPs on biofilm formation was evaluated in comparison with uncharged NPs and control groups (not exposed to NPs) to elucidate the physiological response of the pathogen to the surface charge of NPs. To our knowledge, this study is one of the first to show the influence of the surface charge of NPs on biofilm formation by pathogenic *E. coli* O157:H7 strain on MP fragments with evidence from analysis of cellular transcriptomics and gene expression.

## Materials and methods

### Materials

All the experiments were conducted using Pyrex glassware obtained from Fisher Scientific (Waltham, MA, USA) or VWR (Radnor, PA, USA). Culture media and reagents were obtained from Sigma-Aldrich (St. Louis, MO, USA) and Bio-Rad (Hercules, CA, USA), if not mentioned otherwise. Polystyrene-based nano-sized spherical beads:**1** Amine-modified^+ve^ (size range, 30–100 nm), **2** Carboxylate-modified^−ve^ (size range:30–100 nm), **3** Uncharged (size range, 30–100 nm) were obtained from Sigma-Aldrich and Polysciences, Inc. (Warrington, PA, USA). Polyethylene terephthalate fragments (cat. no. LS561482, thickness 0.006 mm, length 500 mm, width 315 mm) were obtained from Goodfellow Corporation (Pittsburgh, PA, USA). FilmTracer FM 1–43 green biofilm cell stain (cat no. F10317) was obtained from Invitrogen (Thermo Fisher Scientific, Waltham, MA, USA). PCR primers used in the experiments were obtained from Sigma-Aldrich.

### DLS and zeta (ζ) potential of NPs

The hydrodynamic diameter, polydispersity index (PDI), and stability of the NPs in aqueous suspension were estimated using dynamic light scattering (DLS) and ζ-potential measurements [20, 21]. For analysis NPs were suspended in deionized (DI) water at 25 °C in a Malvern Zetasizer Nano ZSP series. Each sample was measured in disposable folded capillary micro-cuvettes three consecutive times with a 2 s delay between measurements. The duration of the measurements varied from 50 to 100 s depending on the dispersity of the sample.

### Bacterial strain and growth condition

A rifampicin-resistant pathogenic *E. coli* O157:H7 strain EDL933 (ATCC 43895) was used in all experiments. The strain was sub-cultured on Cefixime-Tellurite Sorbitol-MacConkey (CT–SMAC) agar plates. For experiments, EDL933 cells were grown overnight at 37 °C and 200 rpm in LB broth supplemented with 100 μg/mL of sterile rifampicin, followed by re-inoculation in LB broth (0.1% v/v) and incubation at 37 °C and 200 rpm for 3–4 h to obtain mid-log phase bacterial cells (OD_600nm_ = 0.6; 10^6^ CFU/mL) to start all experiments.

### Bacterial viability and growth kinetics

The growth and viability of *E. coli* O157:H7 cells (control) and exposed to NP(0), NP( +), and NP(−) at two different concentrations (50 and 100 mg/L) was studied up to 7 days in LB broth by monitoring the gradual increase in optical density (OD_600 nm_) and plate-based counting using the serial dilution method. Sterile LB broth without any bacteria and NPs served as a negative control, while *E. coli* O157:H7 culture in LB broth without any NPs served as a positive control. LB broth with NPs of three different types used for the experiments without any inoculation of bacteria, but incubated under the same experimental conditions, served as respective experimental blanks for each treatment. The difference in OD_600 nm_ for each blank and test (both in triplicate) were recorded at 1 h interval up to 24 h with a LabSystems Bioscreen C microplate absorbance reader (Oy Growth Curves Ab Ltd., Finland). Changes in OD_600 nm_ with respect to the blank represented the changes in growth pattern, which were compared for the positive control and three test samples upon exposure to NPs. A modified Gompertz growth model [22] was used to fit all the growth curves (control and treated with NPs). The lag time, specific growth rate, and generation time of bacteria with and without exposure to NPs were estimated from the fitted equation parameters. The viable plate count of *E. coli* O157:H7 was recorded at several intervals up to 7th day of incubation for planktonic cells, using the plate-based assay on BHI agar, incubated at 37 °C for 24 h.

### Biofilm formation on microplastic

The growth of biofilms by *E. coli* O157:H7 on the surface of 6 µm thick PET-based MP fragments (~ 50 × 50 mm^2^) was studied at 7- and 15-day. The PET fragments were sterilized with 100% ethanol (soaked for 20 min), followed by air drying under sterile conditions prior to use in the biofilm growth experiments. Glass petri-dishes (60 × 15 mm^2^) were used for each experiment, where 5.0 mL of LB-broth was added to submerge one PET fragment per petri-dish. A 0.1% inoculum (OD_600 nm_ 0.6) was added to the LB broth in Petri dishes, apart from the negative controls for all treatments (containing PS-NPs but no *E. coli* O157:H7). Four sets of plates (one set as positive control and three sets of treatments) were incubated undisturbed under static conditions at room temperature. Biofilm growth of *E. coli* O157:H7 on PET-based MP fragments (not exposed to NPs) served as the biofilm control, and treatments included 100 mg/L of NP(0), NP( +), and NP(−) supplemented to the LB broth. Separate plates were incubated at room temperature under static conditions for 7- and 15-days.

For microscopic visualization, simultaneous biofilm growth experiments were started in separate petri dishes containing several small PET fragments (~ 10 × 10 mm^2^), inoculated, and incubated under the same conditions for all treatment groups.

#### Dissociation of biofilm

Biofilm was dislodged from PET fragments (50 × 50 mm^2^) on 7- and 15-day, by repeated washing with 3 mL of 0.85% NaCl and collecting the suspension in sterile glass petri-dishes (60 × 15 mm^2^). 1.0 mL of the bacterial cell suspension was separated out and immediately used for assessment of bacterial cell viability through both plate-based assay and flow-cytometry assay. Rest of the cell suspension was used for biochemical estimation of carbohydrate and protein content in biofilm samples and measurement of oxidative stress in biofilm cells. Cell-free extracts were prepared by vigorous shaking of bacterial cell suspensions (eight times for 20 s, under cold conditions) with 0.5 mm glass beads (E.Z.N.A. Omega Biotek). Supernatant was then collected by centrifugation (1000 × g for 10 min) at 4 °C followed by storage at − 80 °C until experimentation, as described previously [23].

### Assessment of biofilms by confocal laser scanning microscopy (CLSM)

After 7 and 15 days of biofilm formation, the PET fragments (~ 10 × 10 mm^2^) were carefully transferred to clean glass microscopic slides using a sterile stainless-steel forceps, air-dried for 30 min, washed with sterile DI water, and stained with 1 µg/mL FilmTracer^™^ FM^™^ 1–43 Green Biofilm Cell Stain (Invitrogen, cat no. F10317), following the manufacturer’s instructions. Briefly, the samples were incubated in the dark for 30 min after the addition of the dye, and then washed twice with sterile water to clear any background stain. The samples (biofilm on PET fragments) were fixed individually on microscopic slides by mounting a coverslip on top and visualized by a Zeiss LSM 710 CLSM with a 63X oil immersion objective. Z-stack images were acquired under optimized settings (excitation/emission at 472/580 nm, Gain 700, and pinhole 1.0 AU, z step-size 0.1 μm) to capture detailed biofilm structures. Three-dimensional images were reconstructed and biofilm biovolume was estimated from the confocal z-stacks using Bitplane Imaris imaging software (v. 10.1.1) [24–26].

Biofilm growth was also quantified using semi-quantitative crystal violet (CV) assay. After incubation for specific period, the media was aspirated, and biofilms were air-dried for 30 min. The petri dishes were then rinsed with deionized water to remove non-adherent cells before staining with 100 µL of 0.1% (m/v) CV for 15 min at room temperature. Excess CV was washed off with DI water, and biofilms were decolorized with 200 µL of 90% (v/v) ethanol for 3 min. Finally, about 125 µL of the extracted solution was transferred to a 96-well plate, and absorbance was measured at 595 nm using a multifunctional plate reader (Multiskan Ascent, Thermo Electron Corporation) [27, 28].

### Scanning electron microscopy

An environmental scanning electron microscope (ESEM) with a field-emission electron gun (Quanta FEG 450) was used to capture images of the three types of NPs used in this study. The interactions of planktonic cells with NPs and biofilm growth on the surface of the PET fragments were visualized using ESEM micrographs. After exposure to the NPs, planktonic cells were harvested and washed twice with PBS (pH 7.0), followed by treatment with 2% glutaraldehyde in 0.1 M phosphate buffer (pH 4.0), for a minimum of 4 h at 4 °C [2]. Similarly, PET fragments after rapid washing with sterile water, were submerged in 2% glutaraldehyde buffer (pH 4.0) for 4 h at 4 °C. The planktonic cells and PET fragments were washed twice with 0.1 M phosphate buffer (pH 4.0), followed by alcohol gradation treatment (10%, 25%, 50%, 70% and 100%) [29]. For planktonic cells, dried powdered samples were directly sprinkled over the stubs with carbon tape. After proper drying, the PET fragments were mounted on stubs with carbon tape. To avoid charging during imaging, a thin coating of gold/palladium (~ 10 nm) was evaporated on top of the samples (using a Denton TSC turbopumped sputter coater). Images were acquired under a high vacuum using a landing voltage of 20/30 kV, spot size of 3.0, pressure of 5.60e^−5^ Torr, and working distance of 10 mm. Different magnifications from 16000 to 30000X were used for different samples to locate bacteria or NPs.

### Carbohydrate and protein estimation of biofilms

Carbohydrate and protein contents were estimated using the anthrone [30] and Bradford method [31], respectively. The cell supernatant was treated with anthrone (Sigma, cat no. 52445-100MG) and Bradford reagent (Sigma, cat no. B6916-500ML) followed by spectrophotometric measurements (Thermo Scientific Biomate 3). Carbohydrates present in the cell cytosol are dehydrated using concentrated H_2_SO_4_ (Anthrone reagent) to form furfural, which then condenses with anthrone to produce a green color that was estimated spectrophotometrically at λ_max_ 620 nm. Bradford assay is based on the absorbance shift (λ_max_ 595 nm) of the dye Coomassie brilliant blue, depending on the amino acid composition of the protein in the sample. The standard curve for carbohydrate estimation was measured with glucose (stock concentration 1.0 mg/ml), while that for protein was measured with bovine serum albumin (stock concentration 2.0 mg/ml).

### Flow cytometry

The viability of planktonic bacterial cells was measured after incubation at 37 °C, 200 rpm at 24 h and 7-day, and for biofilm samples, at 7- and 15-day, using a Live/Dead BacLight Bacterial Viability and Counting Kit (Invitrogen, cat no. L34856). Bacterial cells were prepared for the assay following protocols described in the kit. To distinguish and quantitate the live and dead bacteria a BD FACSymphony A1 flow cytometer, equipped with a laser emitting at 488 nm was used, and data processed with BD FACSDiva 9.0.2 software. 3.34 mM SYTO 9 nucleic acid stain was used to stain live cells, whereas 30 mM propidium iodide was used to stain dead cells. Fluorescence was collected in the green and red channels using appropriate filters. The gating was done based on compensation control experiment to set the forward scatter and side scatter fluorescence signal, which was used in all the experimental sets. During flow cytometry analysis of samples, a 6.0 μm diameter microsphere standard supplied with the kit was included in each flow cytometry analysis tubes to quantify the number of bacteria per mL [32]. The protocol used as per the kit is described in the flow cytometry section of supplementary file.

### Malondialdehyde assay

MDA production was measured according to a previously described method [33] with little modifications. Briefly, 0.5 mL of bacterial supernatant was mixed thoroughly with 1.0 ml of TBA-reagent (15% w/v trichloroacetic acid, 0.375% w/v thiobarbituric acid; 0.25 N hydrochloric acid), heated for 15–20 min in a boiling water bath, cooled, and the supernatant was collected by centrifugation (1000 × g, 10 min) at 4 °C. The absorbance of the sample was measured at 535 and 600 nm. The MDA equivalent content in the sample was calculated using an extinction coefficient of 1.56 × 10^5^/M/cm.

### Catalase assay

Catalase activity in the bacterial supernatant was measured following the method described [34] with little modification. In brief, 20 μl of supernatant was mixed with 980 μl of 30 mM H_2_O_2_-phosphate buffer and the rapid decomposition of H_2_0_2_ was measured as the difference in absorbance (∆A_240_) per unit time using a spectrophotometer for 60 s at 15 s intervals. The rate constant (k) for the overall reaction is given by K = (2.3/∆t) (logA1/A2), where K is the rate constant of the first-order reaction, ∆t is the difference in time = t_final_ − t_0_, A1 is A_260_ at t = 0, and A2 is A_260_ at t = final time.

### RNA extraction and quality assessment

Bacterial RNA was extracted separately from all samples, following the manufacturer’s protocol described in the RNeasy^®^ PowerFecal^®^ Pro Kit (Qiagen, cat no. 78404). After extraction, the purity, integrity, and concentration of these RNA were checked using Nanodrop and Qubit (Thermo Scientific). The quality was assayed by 1% agarose gel electrophoresis (operated at 70 V for 60 min) using a 1 kb DNA Ladder (New England Biolabs, cat no. N3232S). RNA integrity was checked using a 5200 Fragment Analyzer (Agilent Technologies, Santa Clara, CA, USA). RNA samples were stored at − 80 °C until further use.

### Sequencing and bioinformatics analysis of RNA-seq data

Library preparation and RNA sequencing were performed according to the methods described [35] with little modifications. RNA-seq libraries were prepared using the Kapa Hyper Stranded RNA kit (Roche). Ribosomal RNA was removed using the Qiagen Fastselect Bacteria kit (Cat No. 335927). The libraries were pooled, quantitated by PCR, and sequenced on one SP lane for 151 cycles from both ends of the fragments on NovaSeq 6000. Paired-end (2 × 150 bp) sequencing was performed at a depth of 40 million reads for each sample. The adapters were removed, and a quality check was performed using FastQC software. Based on quality check statistics, low-quality reads (q < 25) were removed the FastP software [36]. The clean reads were then aligned and mapped in Salmon’s (v1.10.1) against the transcriptome profile of the * E. coli* O157:H7 strain to obtain quant files [37]. The mapped reads obtained from the quant files were normalized using the count per million (CPM) method with a threshold value of 0.5, followed by log transformation using the EdgeR package [38]. The differential expression of genes was estimated using DESeq2 with ad.P value at 0.05, and a minimum fold change of 2 [39]. Enrichment analysis was performed using GAGE software with a pathway significance cutoff of 2 and minimum gene size of 5 [40].

### Real-time PCR

Bacterial RNA after quality check was subjected to cDNA synthesis using a GoScript Reverse Transcription System (Promega, cat no. A5001) on a T100 Thermal Cycler (Bio-Rad). No reverse transcriptase control (NRTC) was included for each DNase-I-treated RNA sample while cDNA synthesis was performed. qPCR was conducted in a thermocycler (Bio Molecular Systems, Model: mic, S/N: M0004167) using SYBR Green FastMix (Quantabio, cat no. 95071-012) to obtain cycle threshold (CT) values. Following similar protocols and appropriate annealing temperatures for the respective primers, CT values were obtained for 14 target genes of *E. coli* O157:H7: virulence and adhesion-factor genes (*stx*_*1a*_*, stx*_*2a*_*,* and *eaeA*), stress-response genes (*rpoS,* and *oxyR*), and biofilm formation genes (*fliA, fimH, flhD, crl, chpB, motA, fliC, luxS,* and *bolA*) for all samples (no-NP and PS-NPs exposed—24 h planktonic cells, 7-day and 15-day biofilm cells). Gene expression was normalized using three housekeeping genes (*16SrRNA, gapA,* and *mdh*). The data sets were then analyzed using the 2^−ΔΔCT^ method to quantify relative gene expression, and the data are presented as mean log_2_fold-change (for each target gene with respect to three housekeeping genes). All experiments were conducted in triplicate. The specific functions of all the genes studied are reported in Table S2 with primer sequences.

### Statistical analysis

One-way analysis of variance (ANOVA) with the Tukey–Kramer multiple comparisons test was performed using GraphPad Prism 10.2.0. All data are expressed as mean ± standard deviation (SD) of three independent experiments, unless indicated otherwise. For relative gene expression analysis data, the Tukey–Kramer Multiple Comparisons Test was performed between the control and each treatment group. Statistical significance levels were defined as extremely significant (p < 0.001, ***), very significant (p < 0.01, **), and significant (p < 0.05, *).

## Results

### Characterization of PS-NPs

**Fig. 1:**
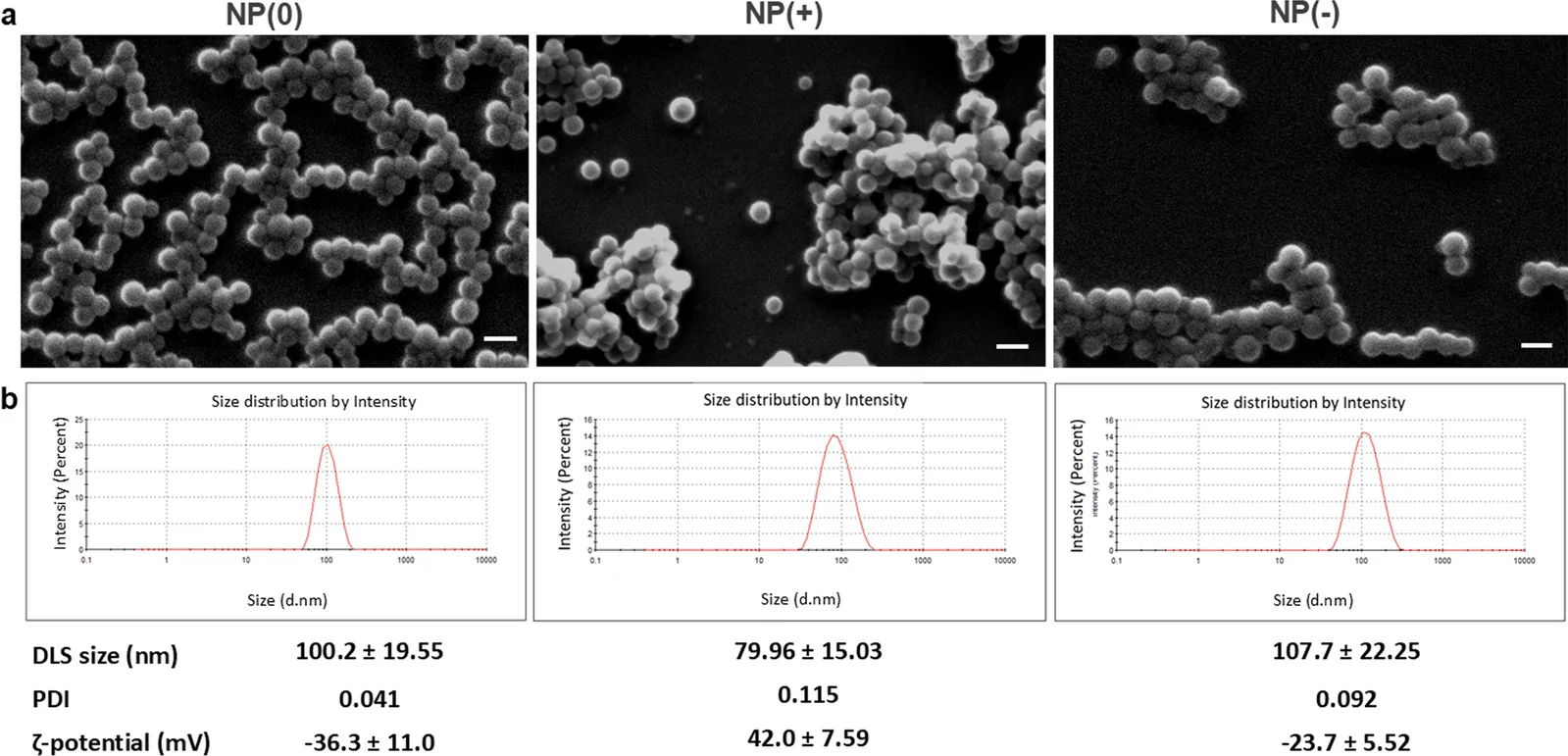
Shape and size distribution of PS-NPs used in this study. **a** Scanning electron micrographs of NP(0), NP( +), and NP(−); scale bars represent 100 nm. **b** Dynamic light scattering (DLS) analysis showing hydrodynamic size distribution of the respective NPs (dispersed in DI-H_2_O at 25 °C); DLS size (nm), Polydispersity index (PDI), and ζ-potential values (mV) for three types of NPs are shown in respective panels

In this study, we characterized the differentially charged NPs—NP(0), NP( +), and NP(−), using SEM, DLS and zeta (ζ) potential measurements, as presented in Fig. 1. SEM images (Fig. 1a) show the morphology of NPs. Intensity versus size peaks from DLS measurements (Fig. 1b) shows hydrodynamic size range of the respective NPs suspended in DI water at 25 °C. The particle size determined by DLS measurement showed size variation from SEM images, as DLS indicates hydrodynamic diameter of dispersed NPs in suspensions, which depend on the thickness of the hydration/solvation layer, thus varying from the core particle size [20, 41]. Polydispersity index (PDI) shows heterogeneity of particle sizes in all three NP samples. Both the SEM images and DLS measurements confirmed the size range of the NPs (≤ 100 nm). The stability of NPs was assessed by measuring the zeta potential of the particles suspended in deionized water at 25 °C. The zeta potential values in Fig. 1, suggest that the NPs were well dispersed in DI water at 25 °C. Zeta-potential is a measure of the magnitude of electrostatic or charge repulsion/attraction between particles which plays an important role in the aggregative stability of particles of nanosizes dispersed in any liquid [42–45]. The identity of the NPs was confirmed using infrared spectroscopy (Fig. S1). The spectra matched well (> 95%) with the polystyrene reference contained in the Hummel Polymer Database.

### Viability and growth kinetics of planktonic *E. coli* O157:H7, upon exposure to surface-charged NPs

**Fig. 2:**
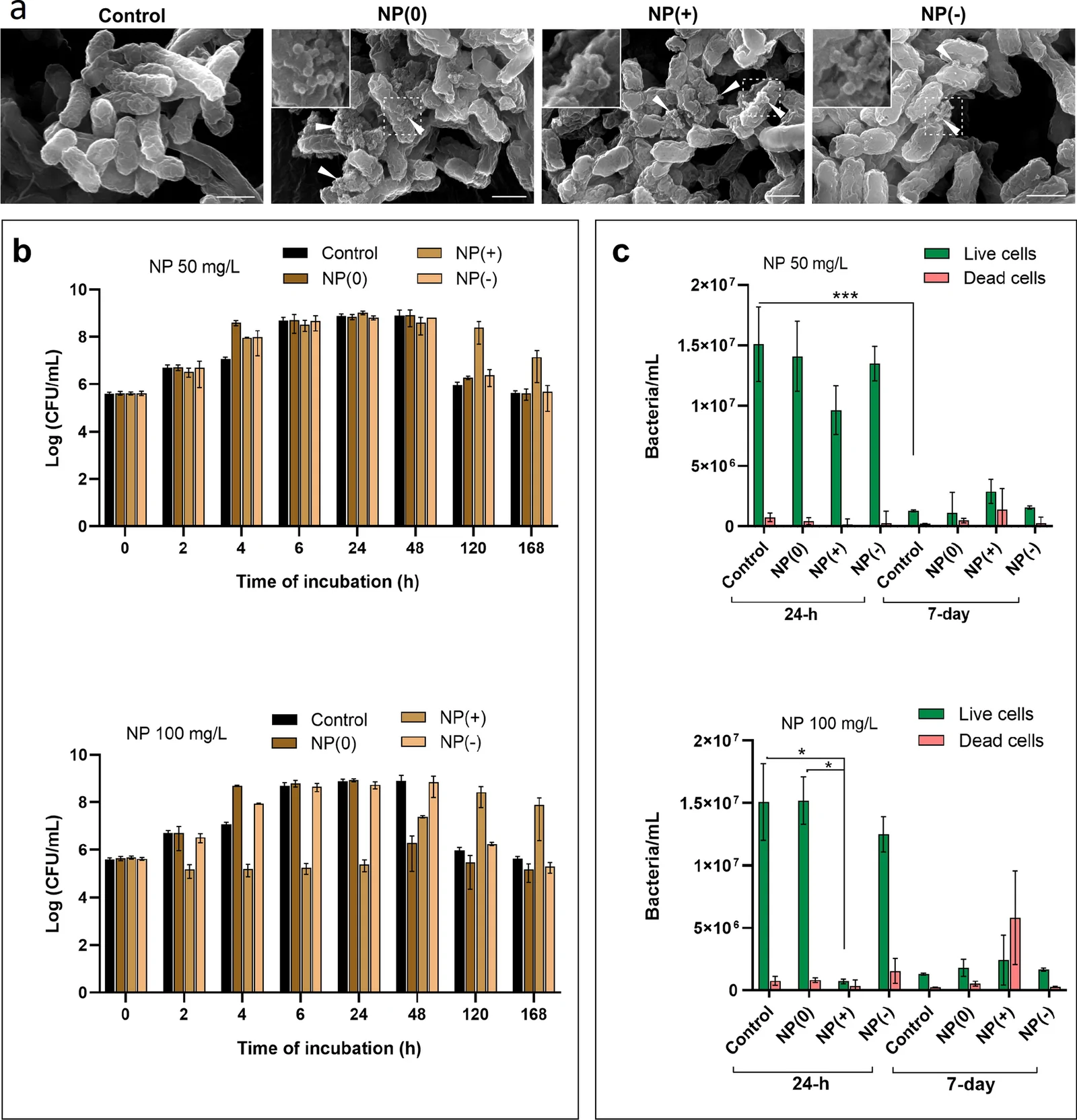
Interaction of planktonic *E. coli* O157:H7 with differentially charged NPs. **a** Scanning electron micrograph showing the interaction of *E. coli* with the respective NPs (white arrows); magnified view of selected area shown in small squares; scale bars represent 1 μm. **b** Viable plate counts of bacteria following exposure to NPs at 50 mg/L and 100 mg/L, measured for up to 7 days (control represents no NP treatment). **c** Flow cytometry analysis of total bacterial load (live/dead) for planktonic *E. coli* treated with NPs at 50 mg/L and 100 mg/L. The cultures were incubated at 37 °C and 200 rpm at 24-h and 7-day, washed, and suspended in 0.85% NaCl (O.D. adjusted to 0.6) prior to analysis. The data represents mean and standard errors for triplicate experiments, * denotes significance at p < 0.05, and *** denotes p < 0.001

The clustering and binding of the respective NPs on the *E. coli* O157:H7 cell surface are shown in the SEM images (Fig. 2a). Both NP(0) and NP( +) were observed to be bound to the bacterial surface as clusters, whereas NP(−) was rarely found to bind to the bacterial surface and was mostly scattered and sparse. Changes in the growth pattern of *E. coli* O157:H7 upon exposure to differentially charged NPs (all similar in size range) at two different concentrations (50 mg/L and 100 mg/L) were monitored for 24 h at 37 °C and 200 rpm, and the results are presented in Table 1. The exposure concentrations were selected based on an optimization experiment, where the growth of the bacteria was studied in the presence of NP( +)s at different concentrations (10, 25, 50, 75, 100, 125, and 150 mg/L) and were found to be concentration dependent (Fig. S2). NP( +) at concentrations higher than 50 mg/L were found toxic to cell growth and had bacteriostatic effects. Based on this optimization, we selected two concentrations (50 and 100 mg/L) for all NP treatments, which is consistent with several other studies focusing on the interaction of MPs/NPs with bacteria, specifically *E. coli* [6, 29, 46].

**Table 1:** Growth kinetic parameters of planktonic *E. coli* O157:H7 (control and NP-exposed) observed for 24 h

*E. coli* O157:H7		Estimated parameters			Fitted equation coefficient (*R*^*2*^)
Treatment	Concentration (mg/L)	Lag time (h)	Growth rate (h^−1^)	Generation time (h)	
Control	0	0.899 ± 0.215	0.26 ± 0.05	0.475 ± 0.084	0.926
NP(0)	50	1.138 ± 0.047	0.23 ± 0.01	0.535 ± 0.019	0.976
	100	0.973 ± 0.44	0.23 ± 0.016	0.535 ± 0.039	0.98
NP( +)	50	2.024 ± 0.39	0.167 ± 0.014	0.725 ± 0.064	0.99
	100	8.982 ± 1.964	0.0365 ± 0.003	3.306 ± 0.217	0.866
NP(−)	50	1.067 ± 0.258	0.216 ± 0.029	0.565 ± 0.073	0.963
	100	1.478 ± 0.248	0.243 ± 0.078	0.536 ± 0.19	0.977

A modified Gompertz sigmoidal growth model [22] was used to estimate the growth parameters, that is, lag time, specific growth rate, and generation time, for the control and NP-exposed bacteria (Table 1). A generation time of 0.475 ± 0.084 h was estimated for *E. coli* O157:H7 (control) grown for 24 h. Extended lag phase (generation time of 0.725 ± 0.064 h was observed upon exposure to 50 mg/L of NP( +) and 3.306 ± 0.217 h upon exposure to 100 mg/L of NP( +). The cells exposed to NP( +) took a longer time, to acclimatize and come out of lag phase. They were found to grow actively at ~ 48 h (Fig. 2b). Results indicate an initial growth arrest and toxicity of NP( +)s on the bacteria.

The viability of *E. coli* O157:H7 control and upon exposure to NPs at 50 and 100 mg/L (based on the plate count method) is shown in Fig. 2b. At a concentration of 50 mg/L, cell viability was not affected in the presence of any NPs, while the viability of the initial population of cells was affected when exposed to 100 mg/L of NP( +) (Fig. 2b). To confirm further, the live and dead cell counts of bacteria (control and NPs-exposed) incubated at 37 °C and 200 rpm, were studied with flow cytometry at 24 h and 7-day time points. The results of the flow cytometry analysis of *E. coli* O157:H7 exposed to 50 and 100 mg/L NPs are shown in Fig. 2c. The viability of bacteria exposed to 100 mg/L of NP( +) was lower at 24 h but then increased on the 7-day compared to bacteria exposed to other NPs. Also, the population of dead bacteria was higher upon exposure to NP( +). Thus, based on these observations, we hypothesized that some, but not all, of the initial population of bacteria exposed to NP( +) could overcome the stress and start to grow after 48 h.

### Biofilm formation by *E. coli* O157:H7 on PET MP in the presence of surface-charged NPs

**Fig. 3:**
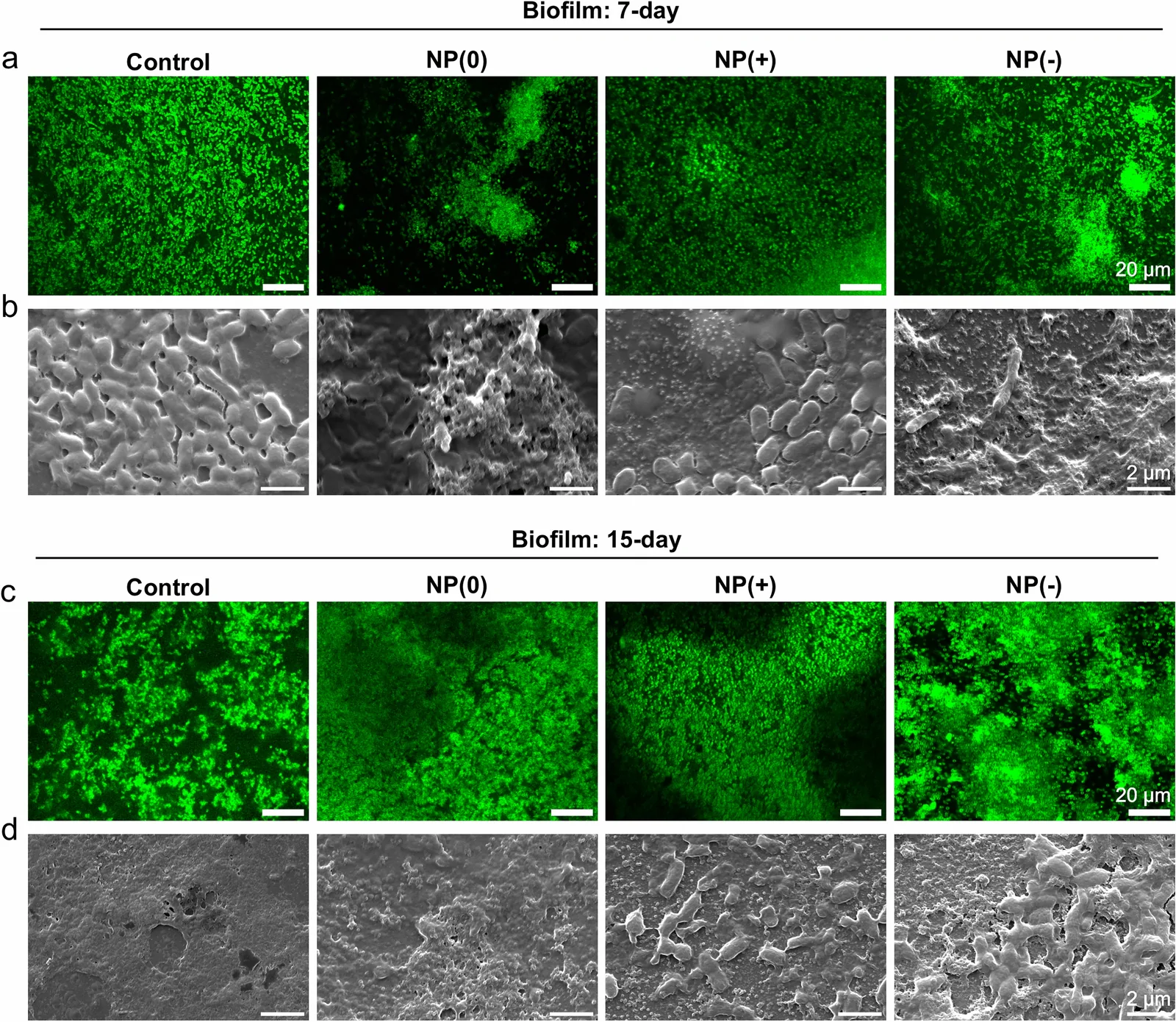
Observation of biofilm development on the surface of PET-based MP fragments (thickness 6 μm) at 7- and 15-day under control condition (no treatment with NPs) and in the presence of 100 mg/L NP(0), NP( +),or NP(−). **a**, **c** Confocal laser scanning microscopy (CLSM) images of biofilm development (stained with green biofilm cell stain; excitation/emission at 472/580 nm, gain 700, and pinhole 1.0 AU) at 7-day (**a**) and 15-day (**c**) for the respective NPs (scale bars represent 20 μm). **b**, **d** Scanning electron micrographs of biofilm formation observed on 7-day (**b**) and 15-day (**d**) at 16000X magnification, spot size 3.0, pressure 4.28e^−5^ Torr, and electron beam intensity 30 kV (scale bars represent 2 μm)

Biofilm formation of *E. coli* O157:H7 on the surface of PET-based MP fragments was observed on 7- and 15-day with no NPs (served as biofilm control) and in the presence of differentially charged NPs (concentration 100 mg/L). CLSM images of biofilm at 7-day (Fig. 3a) in the presence of respective NPs (marked on top of each image), show a comparison of the bacterial cell aggregating for biofilm formation. The CLSM images of biofilm for 15-day (Fig. 3c) showed advancement in biofilm developed in case of control and NP(0)-exposed conditions. Biofilm formation in the presence of NP( +) and NP(−) in the 15-day samples appeared less developed (Fig. 3c). SEM images show a magnified view of bacterial attachments and more detailed features of biofilm in 7-day samples (Fig. 3b), and in 15-day samples (Fig. 3d). The SEM images further illustrated the impact of surface-charged NPs on biofilm formation; the process proceeded more slowly than in the control condition.

### Quantification of biofilms

**Fig. 4:**
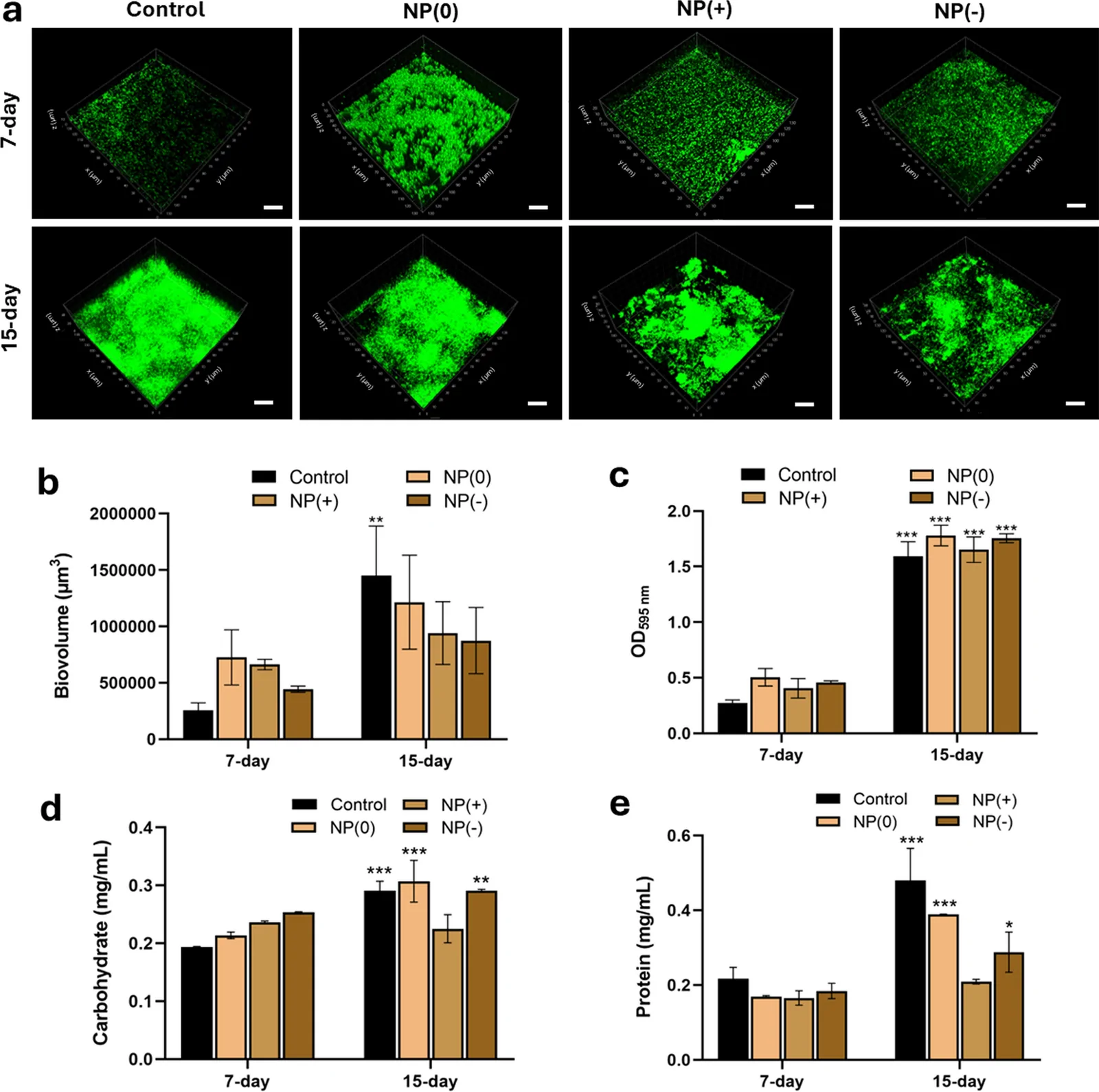
Quantification of biofilm development on the surface of PET-based MP fragments on 7- and 15-day under control (no NP treatment) and in the presence of NP(0), NP( +), and NP(−) at concentration of 100 mg/L. **a** Three-dimensional projections of confocal laser scanning microscopy (CLSM) z-stack imaging of biofilm structures developed under control and NP-exposed conditions (scale bar represents 20 µm). **b** Biovolumes of biofilms (in 16,900 µm^2^ observation field) developed in control and different NP-exposed conditions, quantified from confocal z-stack images using Bitplane Imaris (v. 10.1.1) software. **c** Biofilm growth estimated from crystal violet staining (absorbance at 595 nm) under respective conditions. **d**, **e** Biochemical estimation of carbohydrate content (mg/mL) and protein content (mg/mL) in respective biofilm samples. The data represents mean and standard error of triplicate measurements, * denotes significance at p < 0.05, ** denotes p < 0.01 and *** denotes p < 0.001 for 15-day samples compared against respective 7-day samples

The thickness and three-dimensional structure of the biofilm formed at 7- and 15- days were determined by CLSM z-stack analysis, followed by biovolume estimation in the 130 µm × 130 µm (16,900 µm^2^) observation area using the Imaris software (Fig. 4a and b). CLSM images in Fig. 4a, revealed that the biofilms formed in NP( +) and NP(−) exposed groups were sparse in 7-day samples compared to NP(0) exposed samples (Fig. 4a). The biofilms were thicker and denser in the 15-day samples. A biovolume of 725492 ± 244331 µm^3^ was estimated in the studied observation area in NP(0) exposed 7-day biofilm, while lower biovolumes of 661696 ± 45832 µm^3^ and 443154 ± 27819 µm^3^ were estimated for NP( +) and NP(−) exposed samples respectively. The biovolume was lowest in biofilms not exposed to NPs (control) among the 7-day samples, while the increase in biofilm thickness of the control biofilm on 15-day w.r.t. the 7-day control biofilm was very significant (p < 0.01) (Fig. 4b). The presence of NPs in NP-exposed samples at a concentration of 100 mg/L may have contributed up to a certain level in the biovolume estimation, as biofilms were intact during CLSM analysis, which was also observed in other previous studies [27], which may be the reason for the lower biovolume in the 7-day control compared to the NP-exposed biofilm. Enhanced biovolume was observed in NP-exposed 15-day samples (not significant) compared to the respective 7-day samples. While, NP(0) exhibited less of an effect on biofilm formation, the negative impact of NP( +) and NP(−) on biofilm development is evident in Fig. 4a and b.

Biofilm development was also measured using the crystal violet semi-quantitative method (Fig. 4c). The trend of biofilm thickness observed with CLSM was consistent with the trend observed using the crystal violet method. The absorbance at 595 nm was in the range of 0.4 -0.5 for NP-exposed 7-day biofilm samples, and showed a significant (p < 0.001) increase in respective 15-day biofilm samples, with the highest absorbance observed in NP(0) exposed samples (both 7- and 15-day biofilm) compared to the respective control and surface-charged NP exposed biofilm.

Biofilm growth was also indirectly quantified by estimating the carbohydrate and protein contents in the biofilm matrix at 7- and 15-day. Carbohydrates and proteins are the major constituents of exopolymeric substances (EPS). In addition, the presence of substantial amounts of other substances, such as humic acid, can increase the color intensity during protein analysis using the Bradford method. The biochemical estimation of carbohydrates and proteins in the biofilm samples (7- and 15-day) is shown in Fig. 4d, e, respectively. Under all treatments other than NP( +), the carbohydrate and protein content was found significantly (p < 0.05) higher in 15-day biofilm samples than in 7-day samples, supporting the development of biofilm envelop and progress in biofilm formation, except for NP( +) exposed samples [47–49]. Moreover, in 15-day biofilm samples, the protein content was found to be higher (≥ 0.4 mg/mL) than carbohydrate (⁓ 0.3 mg/mL) in the control and NP(0) samples, whereas in the case of NP( +) and NP(−), contents of carbohydrates and proteins were similar. The higher protein content in the biofilm matrix may be due to the large quantities of exoenzymes entrapped in EPS [50]. In some studies, a greater increase in protein content than in carbohydrates has been associated with the advancement of biofilm formation [47]. Thus, the results of biofilm quantification support the timely progress of biofilm growth on PET-MP fragments for control and NP(0) exposure, but slower development upon exposure to NP( +) and NP(−).

### Viability of cells in biofilms

**Fig. 5:**
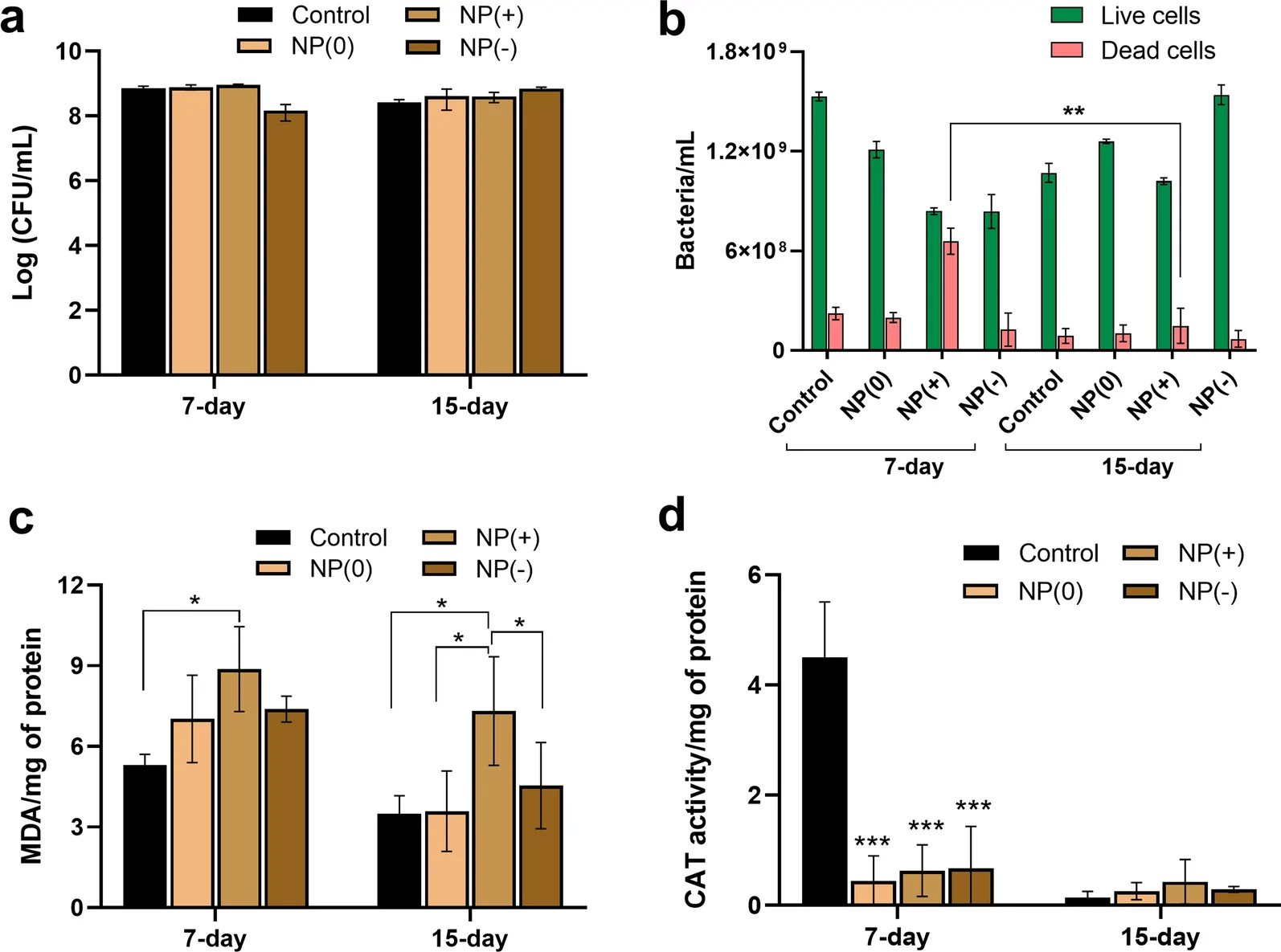
**a** Viable plate counts of bacteria in 7- and 15-day biofilm samples. **b** Live-dead bacterial cell counts estimated by flow-cytometry analysis of 7- and 15-day biofilm samples. **c** Quantification of malondialdehyde (MDA) in 7- and 15-day biofilm samples exposed to differentially charged NPs at 100 mg/L concentration, * denotes significance at p < 0.05. **d** Catalase (CAT) activity in 7- and 15-day biofilm samples, exposed to same set of NPs; *** denotes significance at p < 0.001, compared against the control. The data represents mean and standard error for triplicate experiments

The viability of *E. coli* O157:H7 in the 7- and 15-day biofilm samples was studied using the plate-based method, which showed that in the presence of NP(−), the CFU count in the biofilm sample was lower at 7-day, which increased at 15-day (Fig. 5a). The viability of the bacteria in the 7- and 15-day biofilm samples was further estimated using flow cytometry. Estimation of live and dead bacterial counts showed a relatively lower live cell count in the NP( +)/(−) exposed biofilm at 7-day, compared to the 7-day control (biofilm not exposed to NPs) and NP(0) exposed samples (Fig. 5b), which is probably due to a delay in the initiation of biofilm formation in the presence of charged NPs. Bacterial counts for NP( +)/(−) were found to be higher (not significant) in 15-day flow cytometry estimations. The higher proportion of dead cells in the presence of NP( +) in the biofilm samples could be related to the toxicity of the positive surface charge, as was also found in the case of planktonic cells in this study.

### Oxidative stress in cells forming biofilm upon exposure to surface-charged NPs

Oxidative stress in *E. coli* O157:H7 during the progression of biofilm formation (7- and 15-day) upon exposure to NPs was estimated in terms of MDA levels and catalase enzyme activity (Fig. 5c, d), respectively. The level of MDA was found to increase in both 7-day and 15-day biofilm samples exposed to NPs compared to the control, with a significant (p < 0.05) increase in NP( +) exposure (Fig. 5c), indicating higher cellular stress. Catalase activity was lower upon exposure to all types of NPs during biofilm formation for both 7- and 15-day, compared to the control (Fig. 5d). Such low levels of catalase have often been related to quorum sensing in biofilm samples.

### Global gene expression analysis of planktonic and biofilm condition

**Fig. 6:**
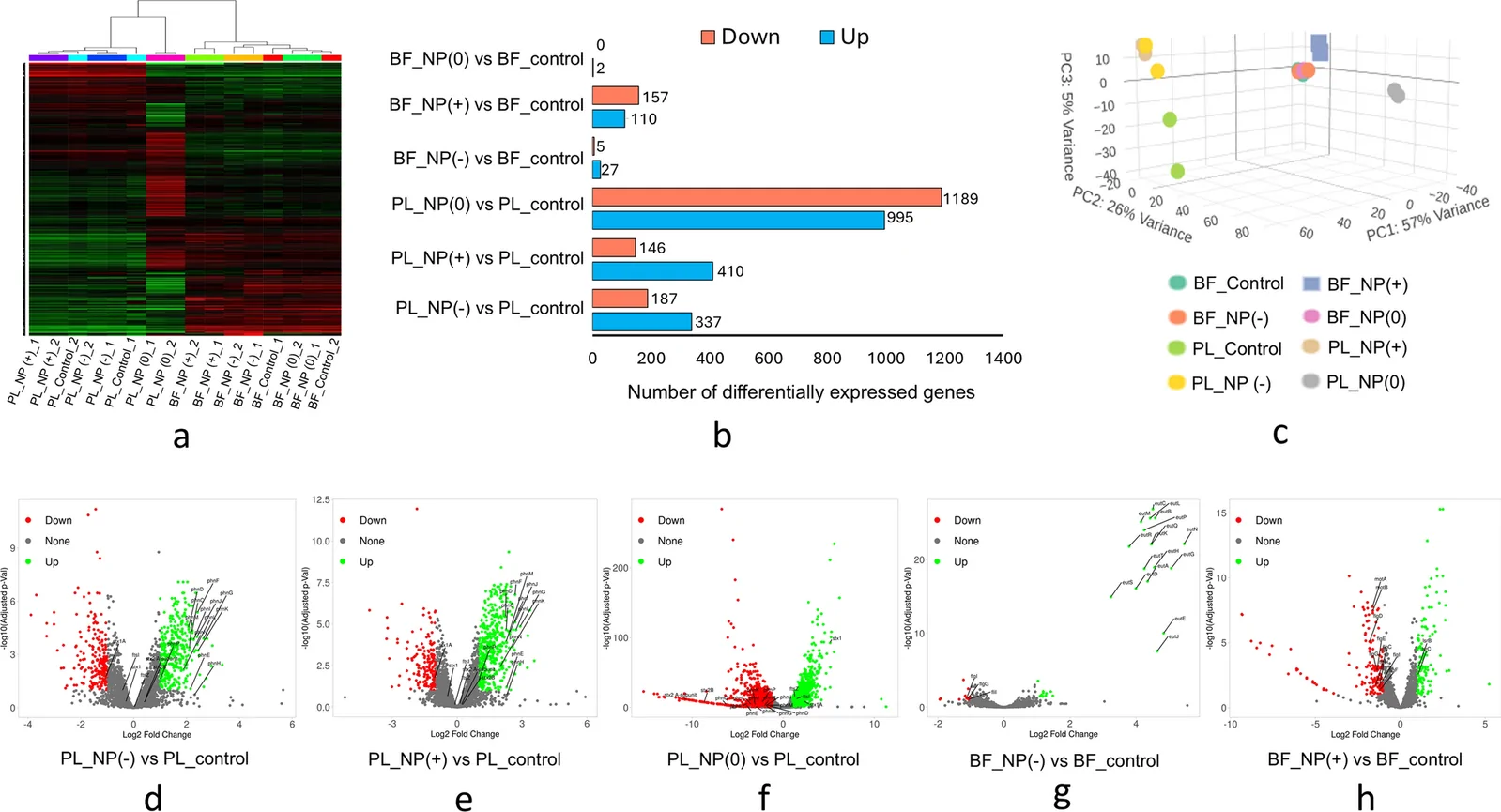
Transcriptomic analysis of the *E. coli* O157:H7 in planktonic (PL) and biofilm (BF) states in the presence of NPs. **a** Heatmap of the expression of genes in different sample groups. Red and green indicate up- and down-regulation, respectively. **b** Number of differentially expressed genes (DEGs) in different NP-treatment groups with respect to the controls (not exposed to NPs). **c** Principal component analysis (PCA) of different sample groups. Clustering of biological replicates from the same sample group indicates a greater similarity between replicates. **d**–**f** Volcano plots of DEGs in planktonic cells exposed to NP(−), NP( +), and NP(0) compared against the PL_control (mid-log phase cells). **g**, **h** Volcano plots of DEGs in biofilm cells (15-day) exposed to NP(−) and NP( +), and compared against BF_control (biofilm not exposed to NPs). Note. Details of key differentially expressed genes are listed in Table S1 of the supplementary file

In this study, the effects of differently charged NPs on the physiology of *E. coli* O157:H7 were assayed under both planktonic (24 h) and biofilm conditions (on PET fragments at 15-day) using transcriptomic analysis (Fig. 6). A comparison of global gene expression patterns among different treatment groups indicated that many genes were either upregulated or downregulated among the different groups (Fig. 6a). The number of differentially expressed genes (DEGs) in the different treatment groups with respect to the control under both planktonic and biofilm conditions is presented in Fig. 6b. The results indicated that biofilms treated with NP(0) had the lowest number of DEGs (2) compared with the biofilm control (BF), whereas planktonic cells treated with NP(0) exhibited the highest number of DEGs (1189 downregulated and 995 upregulated). PCA clearly showed variance in the expression dataset between the planktonic (PL) and biofilm (BF) groups (Fig. 6c). The percentage of variance accounted for by the principal components is shown on the x and y axes. The volcano plots (Fig. 6d–f) explained the statistically significant (padj < 0.05) magnitude of fold change of genes in different treatment groups in planktonic cells compared to the control. For example, cell division-related genes, *ftsL, dksA,* and *ftsZ*, were significantly (padj < 0.05) upregulated (log fold change 0.710553, 0.534819, and 1.275575, respectively) in the PL_NP(0) group compared to the control (Table S1). Furthermore, *phn* operon genes (such as *phnI, phnM, phnF*, etc.) were significantly (padj < 0.05) upregulated in PL_NP(−) (Fig. 6d) and PL_NP( +) (Fig. 6e). However, in the PL_NP(0) group, *stx*2B (log fold change −8.67) and *lom*W (log fold change -11.41) were highly downregulated (Fig. 6f).

In biofilm cells, significant (padj < 0.05) upregulation and downregulation of genes in the different treatment groups, NP(−) and NP( +), are presented in volcano plots (Fig. 6g, h, respectively). Interestingly, NP(0) did not have a significant effect on biofilm physiology compared to planktonic cells (volcano plots not shown, as there were only two DEGs). In BF_NP(−), a series of *eut* operon genes involved in ethanolamine metabolism was significantly upregulated (padj < 0.05), which has a potential role in pathogenicity (Table S1). In BF_NP( +), a series of *lsr* operon genes associated with autoinducer-2 (AI-2)-based quorum sensing, such as *lsrA, lsrB, and lsrC* (log fold change 0.9155, 1.0721, and 1.0434, respectively) were significantly upregulated (padj < 0.05), whereas flagellar assembly genes such as *flgA and flgB* (log fold change −0.9972, and −1.4717, respectively) were significantly downregulated (padj < 0.05) compared to the control (Table S1).

**Fig. 7:**
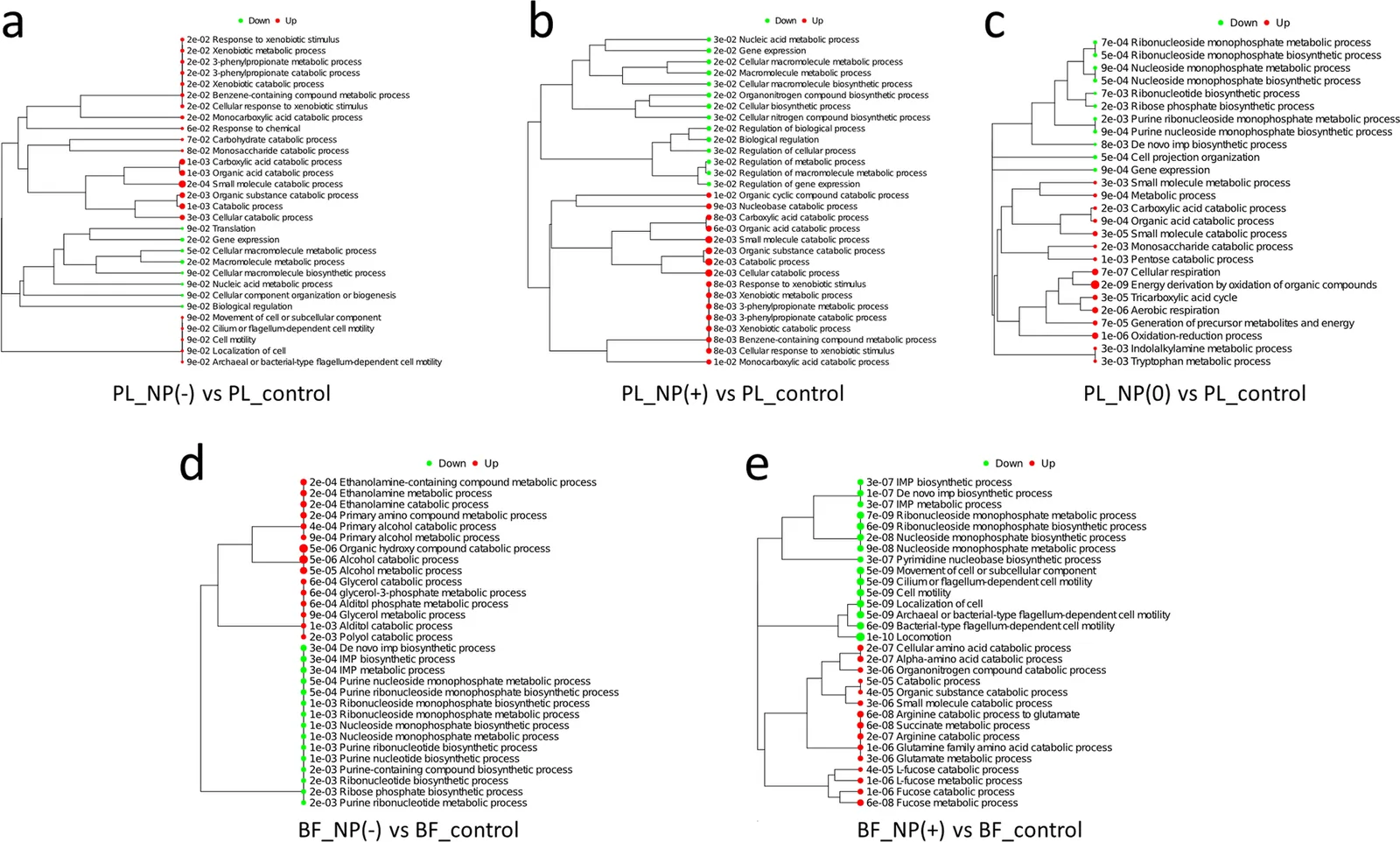
Gene enrichment showing upregulated and downregulated pathways in *E. coli* O157:H7 following treatment with various NPs. **a**–**c** Upregulated and downregulated pathways in NP(−), NP( +), and NP(0)-exposed planktonic cells compared to control (PL_control, mid-log phase cells), respectively. **d**, **e** Upregulated and downregulated pathways in NP(−) and NP( +)-treated groups in biofilm condition with respect to control (BF_control, biofilm not exposed to NPs), respectively. Red and green circles indicate the upregulated and downregulated of pathways, respectively. The numbers designated at each pathway branch represents padj value and indicates the significance of enriched pathways

GO enrichment pathway analysis (Fig. 7) indicated a series of upregulated and downregulated pathways due to changes in global gene expression in the different treatment groups. NP(−) and NP( +) enhanced the upregulation of different metabolic pathways, including 3-phenylpropionate catabolism and xenobiotic catabolic processes in planktonic cells, which are directly related to oxidative stress response, antibiotic resistance, and drug metabolism in bacteria (Fig. 7a, b). Furthermore, several pathogenicity-related pathways such as flagellum-dependent motility and cell localization were upregulated by NP(−) treatment (Fig. 7a). NP(0) induced the upregulation of energy-producing intermediates and pathways; however, most ribonucleoside and purine biosynthetic pathways were downregulated (Fig. 7c).

GO enrichment pathway analysis of biofilm cells indicated that a wide range of biosynthetic pathways (purine, ribose, nucleoside, etc.) were downregulated in the BF_NP( −) group (Fig. 7d). Interestingly, different alcohol and ethanolamine metabolic pathways were significantly upregulated in BF_NP(−). In contrast, in the BF_NP( +) group, a series of pathways related to biofilm formation was downregulated, whereas carbohydrate and amino acid metabolism pathways were upregulated (Fig. 7e). No significant changes were detected in biofilm cells exposed to NP(0) (data not shown).

### Relative gene expression analysis of planktonic and biofilm conditions

**Fig. 8:**
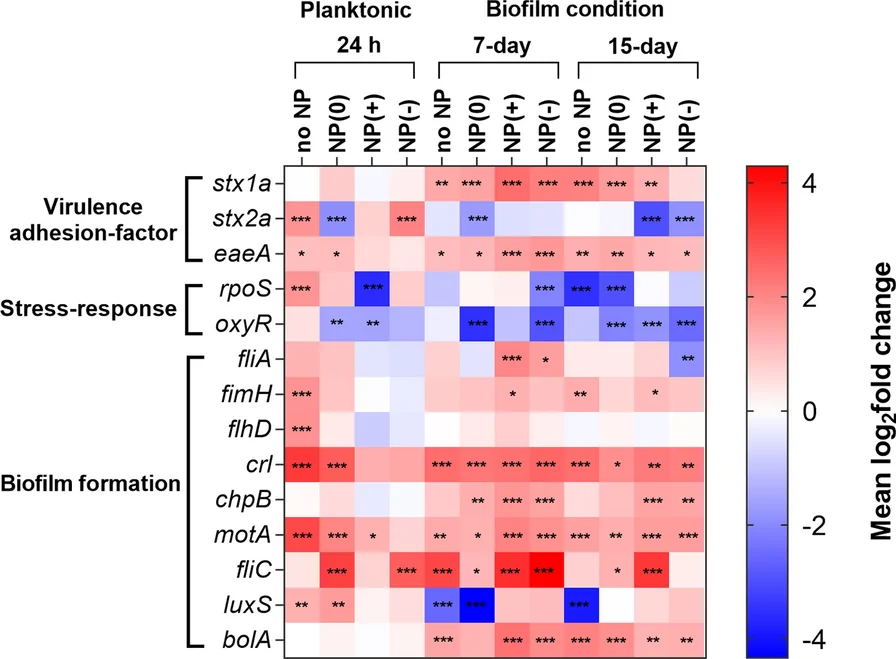
Heatmap of relative gene expression for selected functional genes in *E. coli* O157:H7, quantified through qPCR. Gene expression in mid-log phase *E. coli* O157:H7 cells served as the control for all treatments. Expression of target genes compared to control were normalized using three housekeeping genes (*16SrRNA, gapA, and mdh*). One-way analysis of variance was conducted for statistical comparison between samples (* represents significance p < 0.05; ** represents significance p < 0.01; *** represents significance p < 0.001)

To study the influence of NPs on gene expression, LB broth was supplemented with NPs at a concentration of 100 mg/L and incubated under the same conditions. The differential gene expression of *E. coli* O157:H7 was studied at 24 h for planktonic cells (exposed to NPs) and from biofilm formation on PET fragments (exposed to NPs) at 7- and 15-day. The heatmap showing relative gene expression of selected target genes with respect to the control and normalized to three housekeeping genes is presented in Fig. 8, and the functions of the target genes are summarized in Table S2. Gene expression in mid-log phase *E. coli* O157:H7 cells served as the control for all treatments.

A very significant (p < 0.01) upregulation of the important virulence gene *stx*_*1a*_ was observed in biofilm samples- not exposed to NPs (7-day), while the upregulation was extremely significant (p < 0.001) upon exposure to NPs compared to the control. No significant change was observed for *stx*_*1a*_ in the planktonic cells (24 h exposure). Another important virulence gene, *stx*_*2a*_, was significantly upregulated in NP(−)-exposed planktonic cells at 24 h, but was significantly downregulated upon biofilm formation (log_2_fold-change, −3.0 ± 0.15) at 15-day in the presence of NP(−). The cell adhesion and motility-related genes *eaeA*, *bolA*, *and fliC* were significantly upregulated under biofilm conditions. *fliC* and *eaeA* were also significantly upregulated in planktonic cells following exposure to NP(0) and NP( −). *rpoS*, an important stress response gene, was upregulated in planktonic cells*,* except for NP( +) exposure, where it was significantly downregulated (log_2_fold-change, −3.612 ± 0.251). However, *rpoS* was downregulated under biofilm conditions in all the treatment groups. The stress-response gene *oxyR* was significantly (p < 0.001) downregulated in all treatment groups of planktonic cells and biofilm conditions upon exposure to NP, but not in the no NP exposed biofilm. *crl* and *motA* were significantly upregulated in non-NP and NP(0)-exposed planktonic cells, but not upon exposure to NP(−/ +). In 7- and 15-day biofilm, both *crl* and *motA* were significantly upregulated in the presence of NP( +) and NP(−) exposure. *luxS* was upregulated in no-NP and NP(0)-exposed planktonic, whereas it was significantly downregulated in biofilm conditions. No significant upregulation or downregulation of *luxS* was observed upon exposure to NP( +) or NP(−). The genes *fliA* and *fimH* were significantly upregulated under biofilm conditions in the presence of NP( +). These results indicated changes in the expression of target genes upon exposure to surface-charged NPs.

## Discussion

The surface of Gram-negative bacteria such as *E. coli* is negatively charged due to presence of the highly electronegative groups on lipopolysaccharide (LPS) and other outer membrane lipids [51, 52]. Thus, NP( +) may negatively affect bacterial growth, as observed in growth and viability experiments with planktonic cells in the present study. This finding is consistent with previous studies where the positive charge of PS NPs or other nanoparticles was found to be an important parameter for toxicity against bacterial cells [2, 14, 21, 29, 45]. Likewise, it can be concluded that the slow growth of the bacteria upon exposure to 100 mg/L of NP( +) observed in our study was also due to the toxicity induced by the positive surface charge. At a concentration of 100 mg/L, toxicity of PS-NPs with sizes below 100 nm has been reported by other researchers [6, 29]. The aggregation of positively charged metal-based nanoparticles, which enhance their toxicity to bacterial growth, has been previously reported [8, 53]. The interactions of NPs with bacterial cell walls (and cell membranes), particularly the mechanism governing the entry of the NPs crossing the lipid bilayers of different compositions (Gram-positive vs. Gram-negative) inside the bacterial cells, lacks consensus in the scientific literature [54–56]. However, several electrophysical mechanisms are thought to play key roles in NP-bacterial cell interactions [54, 57–59]. The polarity of the charged particles and lipid molecules (especially LPS in Gram-negative outer membrane) may prevent particles with high net charges from binding strongly (steric hindrance) or passing the membrane barrier of the cells [60]. Thus, charged ( ±) NPs may not enter the cell via simple diffusion [56, 61]. In contrast, uncharged particles readily aggregate on the cell surface and may invade cells via diffusion [55, 62]. This phenomenon, at least in part, may explain the higher number of DEGs in NP(0)-exposed samples than in NP( +) or NP(−) samples observed in our transcriptomic experiments. In this study, we also explored NP( +) concentrations higher than 100 mg/L and found that the exposed cells remained viable for 24 h but showed no growth, indicating a bacteriostatic effect. However, our growth studies show that once the cells exposed to 100 mg/L NP( +) overcame the stress, they resumed growth and remained viable for extended periods. Various molecular mechanisms causing changes in gene expression and physiological alterations, such as ‘growth advantage in the stationary phase’ (GASP) [63], have been proposed to explain the initial growth arrest phenomenon and prolonged viability of bacterial cells under stress conditions [64–68]. It has been demonstrated that bacterial metabolic activities (such as protein synthesis) proceed at a much slower rate in growth-arrested cells compared to the exponential phase, yet these cells can survive for several days to years, and a subpopulation of cells can efficiently resume growth after overcoming stress [64, 69].

Bacterial stress response is an adaptive phenomenon that enables bacteria to survive under various environmental conditions. It is well known that the adsorption or interaction of negatively charged NP with negatively charged bacteria induces stress and can trigger ROS response [9]. MDA is a marker of oxidative stress produced by the membrane lipid peroxidation process caused by different physiological stresses, primarily reactive oxygen species (ROS) [70]; thus, an enhanced level of MDA signifies higher stress levels in cells as found in the present study. However, CAT activity was lower in biofilm samples exposed to NPs. In general, bacterial cells produce catalase to combat environmental stress, which hydrolyzes hydrogen peroxide to water and reduces elevated levels of cellular stress, thereby protecting cell integrity [71, 72]. As reported in a previous study, the catalase activity of *Pseudomonas aeruginosa* in biofilms significantly decreased relative to that of planktonic cells, which was found to be related to quorum sensing. However, despite reduced catalase activity, biofilms were more resistant to hydrogen peroxide treatment than their planktonic counterparts [73, 74]. Another study showed that, in response to silver-NP exposure, catalase activity increased to mitigate stress, while with increasing exposure time, the bacteria lost their ability to detoxify the silver NPs, and subsequently, the catalase activity dropped [75]. In addition to the biochemical assays, we measured the expression of several stress-related genes to better understand the stress physiology of *E. coli* O157:H7 in the presence of differentially charged NPs. We detected a significant downregulation of the *rpoS* gene in NP( +)-exposed planktonic cells (Fig. 8), indicating that *E. coli* O157:H7 was still in the rapid growth phase, which is supported by our growth kinetics results (Fig. 2b, c). However, the 15-day biofilm samples exhibited a downregulation [significant in treatments without NPs and NP(0) groups] of *rpoS*, which might be due to the protection provided by the EPS layer. Studies have reported that the *rpoS*-encoded sigma factor σs controls the expression of several genes involved in cellular responses to a range of stresses, including starvation, osmotic stress, oxidative DNA damage, and transition to the stationary phase, and has been reported to be important for biofilm formation in *E. coli* [76–79]. The regulatory network governing *rpoS* is highly intricate, and our understanding of its transcriptional, translational, and post-translational regulation remains incomplete [80]. Furthermore, a significant downregulation of *oxyR* (which regulates hydrogen peroxide-inducible genes) in the no-NP and NP-exposed cells in biofilm conditions indicates cellular attempts to reduce oxidative stress. Our global gene expression results showed the upregulation of 3-phenylpropionate catabolism and xenobiotic catabolic processes in NP(−/ +)-exposed planktonic cells, indicating cellular stress. In *E. coli*, the positive regulator HcaR, a member of the LysR family of regulators, controls the expression of genes encoding the 3-phenylpropionate dioxygenase complex and 3-phenylpropionate-2′,3′-dihydrodiol dehydrogenase, which oxidizes 3-phenylpropionate to 3-(2,3-dihydroxyphenyl) propionate. These pathways are directly involved in the oxidative stress response [81]. Additionally, upregulation of the *phn* operon, which harbors a set of genes responsible for various stressors [82], in planktonic cells following exposure to NPs, provides evidence of the ability of these cells to manage stress and mount adaptive responses in the presence of NPs. A recent study demonstrated the differential regulation of genes in the *phn* operon in response to zinc oxide nanoparticles (ZnO-NPs) [83]. Their results indicated that ZnO-NPs induced the overexpression of *phnC* and *phnD*, which play an important role in the Pho regulon. Furthermore, previous research has established that the Pho regulon is directly linked to the PhoR/PhoB two-component regulatory system, which controls various virulence activities and stress responses in bacteria including *E. coli* [84].

Biofilm formation enhances bacterial survival by protecting cells against external environmental stress and insults. In our experiment, biofilm formation was found to be affected by the presence of NP( +) and NP(−), which also explains the lower proportions of live cells in NP( +) and NP(−) exposed 7-day biofilm samples, whereas the counts increased in 15-day biofilm samples (Fig. 5b), indicating the progression of biofilm growth. The complex interactions of many genes enable biofilm formation by bacteria, including *E. coli* O157:H7. For example, the gene *chpB*, which is involved in the toxin-antitoxin system (growth inhibitor and suppressor) and is known to contribute to early biofilm formation [85], was also found to be significantly induced. Several adhesion-related genes, including *fliC, fliA, fimH, flhD, crl*, and *motA*, were upregulated in the 7-day biofilm samples across all treatment groups (Fig. 8). Previous studies have highlighted the beneficial roles of these genes during the initial stages of biofilm development [86–90]. Hence, the notable upregulation of these genes unequivocally indicates that exposure to NP(−/ +) leads to slow biofilm formation. In addition to adhesion-related genes, *bolA* expression was markedly upregulated under biofilm conditions in the presence of all PS-NP types. The pleiotropic gene *bolA* is a master regulator of transcription and is associated with several crucial physiological functions in *E. coli*, including biofilm formation, cell motility, adhesion, and curli-fiber formation [91]. Studies have shown that *bolA* plays a crucial role in preserving cell morphology under stressful conditions, and its overexpression can induce biofilm formation [92, 93]. Thus, significant induction of these genes in 7-day biofilms in the presence of NP(−/ +)s may be correlated with the stress response and initiation of biofilm formation, compared to the control and NP(0), which showed good progress in biofilm development. On the other hand, the global gene expression pattern indicated that operons such as *phn* and *lsr* were differentially induced in NP-exposed biofilm conditions (Table S1). For example, the *phn* operon (comprising 14 genes that encode proteins related to the transport and utilization of phosphonates [94, 95]) was also upregulated in planktonic cells exposed to NP(−) or NP( +). The *phn* operon genes are known modulators of biofilm formation and may contribute to biofilm resistance and resilience [96]. The enhanced expression of these genes indicates the initial stages of biofilm formation. A recent global RNA sequencing study indicated the expression of several quorum sensing (QS) related genes, including the *lsr* operon genes in *E. coli* under nanoparticle-induced conditions [97]. Their results indicated that the induction of QS-related genes helps bacteria adapt and survive in nanoparticle-induced conditions. Additionally, studies in *S. typhimurium* have demonstrated that *the lsr* operon genes are involved in the transport and processing of AI-2-phosphate and regulate QS-mediated bacterial behaviors, including biofilm formation and the activation of several virulence-associated factors [98]. The gene expression data also showed a significant upregulation of *luxS* in non-NP-and NP(0)-exposed planktonic cells, suggesting a stationary phase of growth. Previous studies have reported that *luxS* regulates a series of genes associated with different physiological functions, including biofilm formation [99]. Interestingly, AI-2 production was found to be dependent on the presence of *luxS*, and AI-2 accumulation was reported to be highest during the stationary phase and initial biofilm formation [100, 101]. Biofilm formation by Gram-negative bacteria, including *E. coli*, is contingent on cell density [102]. In *E. coli*, the *lsr* operon is suppressed when cell density is low but becomes activated as cell density increases, particularly during the mid-exponential growth phase [103]. Thus, the upregulation of *lsr* operon genes in NP( ±) exposed 15-day samples might positively correlate with the expression of *luxS* under biofilm conditions. In *P. aeruginosa,* enhanced biofilm formation was observed upon PS-NP exposure through upregulation of biofilm-related genes, increased EPS and virulence factor secretion, and enhanced bacterial motility through the participation of the QS system [27]. PS-NPs and magnetite NPs were collectively found to promote biofilm formation in *P. aeruginosa* by stimulating intracellular reactive oxidative species (ROS) production, resulting in the upregulation of QS and GMP signaling pathways and enhanced biosynthesis of polysaccharides [104].

In addition to assessing the expression of stress- and biofilm-related genes, we measured the expression of three critical virulence factors in *E. coli* O157:H7. Our qPCR data showed a significant upregulation of *stx*_1a_ in 7-day biofilm cells exposed to NPs compared to that in the no-NP group (Fig. 8). However, *stx*_2a_ expression was downregulated in most groups under both planktonic and biofilm conditions. Notably, Stx1 and Stx2 are two major classes of Shiga toxins produced by *E. coli* O157:H7 and are known to be induced under stress conditions [72, 105, 106]. The differential expression of these two genes during stress conditions in *E. coli* O157:H7 is common and has been previously reported [90]. The adhesion-related gene *eaeA* was also significantly upregulated under biofilm conditions in the presence of all NPs. Notably, *eaeA* encodes an adherence protein, intimin, in both enteropathogenic *E. coli* (EPEC) and Shiga toxin-producing *E. coli* (STEC) [107–109]. It plays an essential role in the initiation of *E. coli* attachment to the mucosal surface of the host [110] and has been linked to environmental stress [111]. For example, downregulation and upregulation of *eaeA* have been reported during cold storage and heat treatment, respectively [105, 111]. However, global gene expression results showed significant upregulation of the *eut* operon in NP(−)-exposed biofilm cells. The *eut* operon encodes the transcriptional regulator EutR, which helps regulate ethanolamine metabolism and bacterial colonization of the gastrointestinal tract [112] and is thus recognized as a virulence factor [112]. Ethanolamine metabolism is an important characteristic of enteric pathogens, including *E. coli* O157:H7 [112–114], and may affect the virulence of bacteria upon exposure to NP(−). The stress regulatory protein CsrA has been shown to modulate *eut* operon expression in response to environmental stress, allowing bacteria to adapt and survive [115]. Similar to *E. coli*, other foodborne pathogens, such as *Salmonella Typhimurium* and *Listeria monocytogenes*, also utilize the *eut* operon to thrive in environments such as egg yolk [116] and smoked salmon [117]. Therefore, there may be a potential link between nanomaterial-induced stress and activation of the *eut* operon, which warrants further investigation. Therefore, our results indicate that NP( ±) can induce physiological stress, potentially leading to the increased virulence of *E. coli* O157:H7.

## Conclusion

This study with differentially charged PS-based NPs, shows that NPs without surface charge are not acutely toxic to bacterial cell growth and other physiological processes; however, surface charge of NPs adds to their toxicity. The differential physiological (growth and viability) impact on *E*. *coli* O157:H7 upon exposure to charged versus uncharged NPs found in this study underscores the crucial role of surface charge of NPs as a physiological stress inducer in a human pathogen. The stress exerted by the charged NPs initially caused growth arrest (in both planktonic and biofilm cells). Eventually, a subpopulation that overcame the stress, but not all *E. coli* cells, grew unexpectedly later than usual. Gene expression analysis showed significant upregulation of genes encoding a set of stress-response pathways and virulence factors upon exposure to both positively and negatively charged NPs. Therefore, it can be concluded that surface-charged NPs induce physiological stress with a potential risk of increased virulence in this pathogen. Increased survivability of a pathogen and upregulation of virulence genes upon biofilm formation are major concerns with respect to enteric human pathogens such as *E. coli* O157:H7. Thus, in a real environment or ecosystem, where biofilm formation on macro/microplastics and interactions with naturally degraded NPs are unavoidable, such interactions may lead to enhanced survival of pathogens with increased virulence traits.

## Supplementary Information

Supplementary material 1.

## Data Availability

No datasets were generated or analysed during the current study.
