# Correction: Nanoplastics-mediated physiologic and genomic responses in pathogenic *Escherichia* *coli* O157:H7

**DOI:** 10.1186/s12951-026-05128-0

**Published:** 2026-10-08

**Authors:** Jayashree Nath, Goutam Banerjee, Jayita De, Noella Dsouza, Shantanu Sur, John W. Scott, Pratik Banerjee

**Affiliations:** 1 Department of Food Science and Human Nutrition, University of Illinois at Urbana-Champaign, Urbana, 61801 IL USA; 2 Department of Biology, Clarkson University, Potsdam, 13699 NY USA; 3 Prairie Research Institute, Illinois Sustainable Technology Center, University of Illinois, Champaign, 61820 IL USA

**Correction: Journal of Nanobiotechnology (2025) 23:304**

**https://doi.org/10.1186/s12951-025-03369-z**

In this article, an incorrect sub-image (Fig. 3c, last panel [NP(-)]) was inadvertently included in the Fig. 3 image during figure assembly. For completeness and transparency, the old incorrect and correct versions of Fig. 3 are displayed below.

Incorrect Fig. 3.

**Figure:**
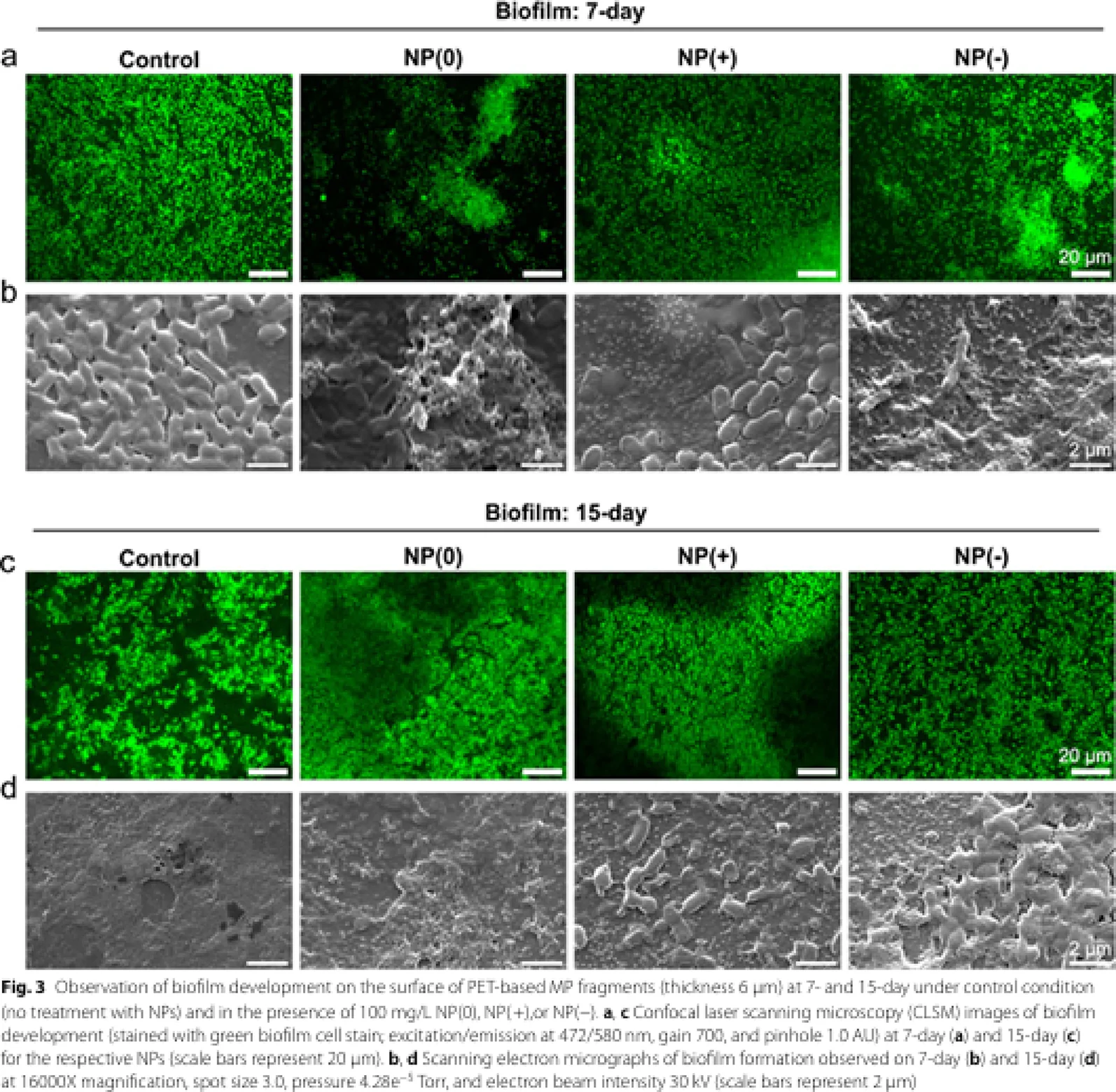

Correct Fig. 3. 

**Figure:**
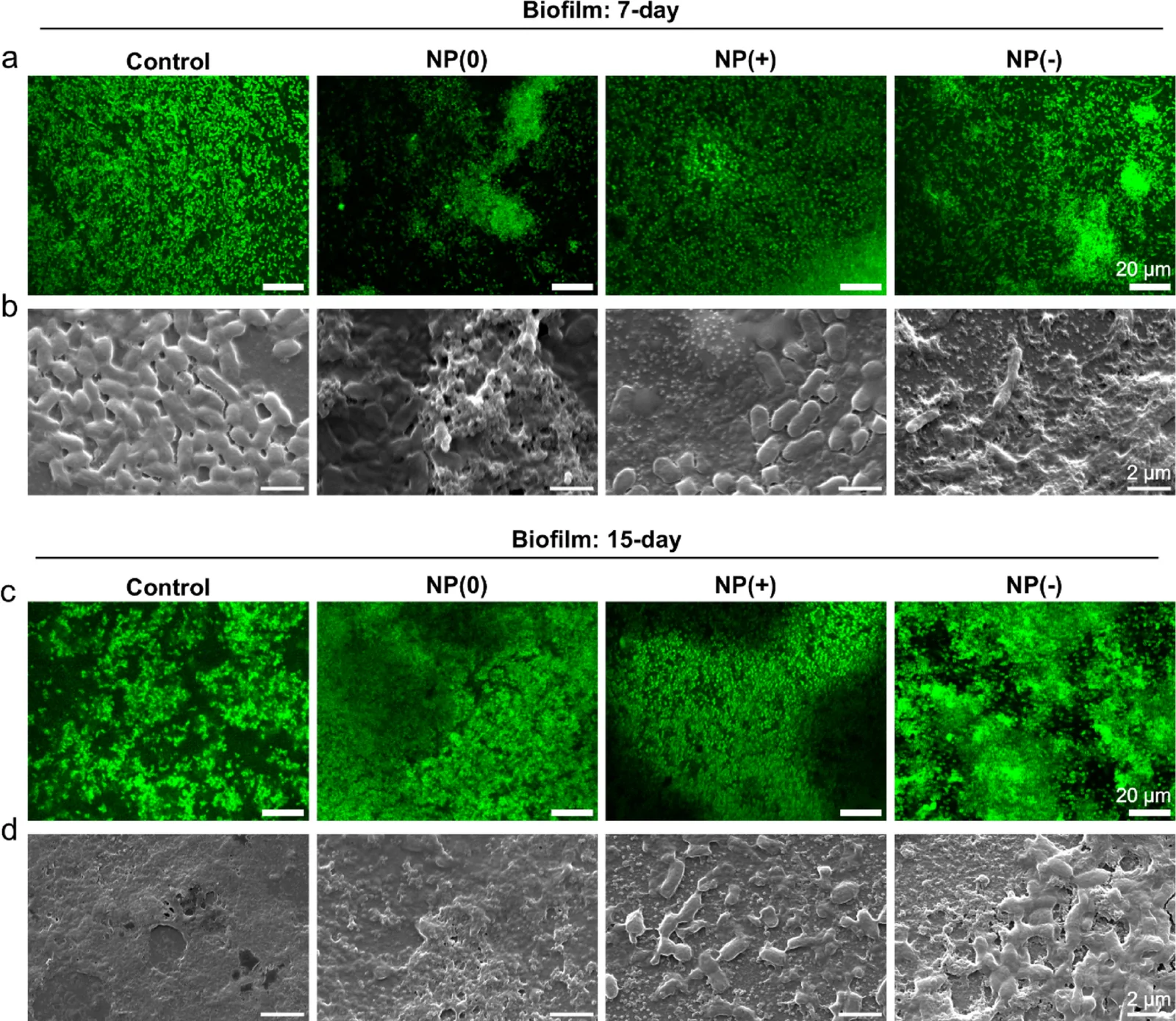

The original article has been corrected.

