# Supplementary material for: Nanoplastics-mediated physiologic and genomic responses in pathogenic Escherichia coli O157:H7
Source: J Nanobiotechnology. 2025 Apr 21;23:304. doi: 10.1186/s12951-025-03369-z (PMC12013119; doi:10.1186/s12951-025-03369-z)
Supplement: Supplementary file 1 — Supplementary material 1. [file 12951_2025_3369_MOESM1_ESM.docx]

Supplementary Information

**Nanoplastics-mediated physiologic and genomic responses in pathogenic *Escherichia coli* O157:H7**

Jayashree Nath^1^, Goutam Banerjee^1^, Jayita De^1^, Noella Dsouza^1^, Shantanu Sur^2^, John W. Scott^3^, Pratik Banerjee^1^*

1. Department of Food Science and Human Nutrition, University of Illinois at Urbana-Champaign, Urbana, Illinois, 61801, USA
2. Department of Biology, Clarkson University, Potsdam, New York, 13699, USA
3. Illinois Sustainable Technology Center, Prairie Research Institute, University of Illinois, Champaign, IL 61820, USA

*Corresponding author: Pratik Banerjee ([pratik@illinois.edu](mailto:pratik@illinois.edu))

**Characterization of PS-based nanoplastics (NPs) and PET-based microplastic fragments used in the study.** ATR-IR spectroscopy confirmed the identity of PS-NPs used in all experiments of the study and PET-fragments used for biofilm experiments (Fig S1).


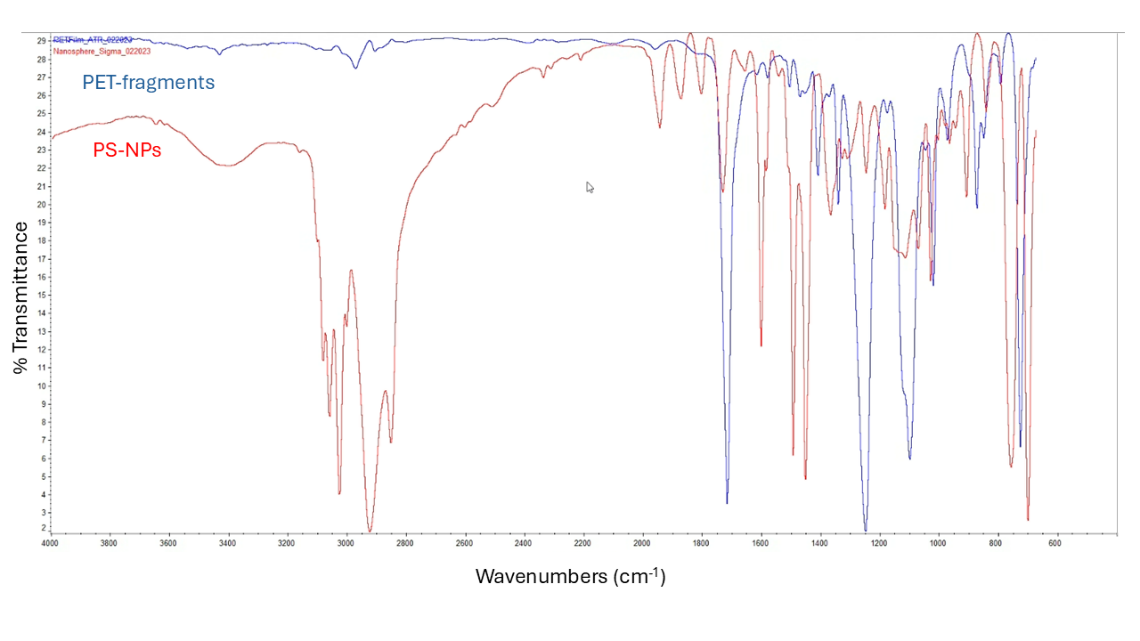


**Fig. S1** Infrared Spectra of PET-fragments and PS-NPs used for bacterial exposure and interaction studies. IR spectra were collected from spot cast on gold coated slides, taken through a Thermo iN10 infrared laser (IR) equipped microscope.


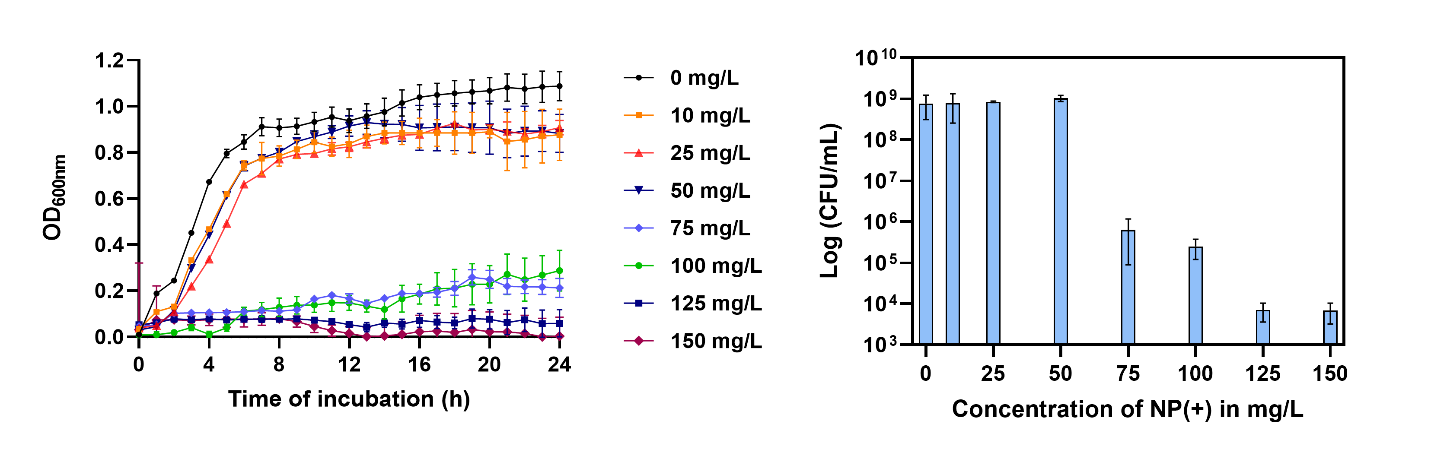


**Fig. S2** **a** Growth curve of *E. coli* O157:H7 (Control – 0 mg/L) and upon exposure to NP(+) at different concentrations (labeled). **b** Viable plate count of the bacteria (control - 0 mg/L) and upon exposure to NP(+) at different concentrations observed for 24 h, 37 °C, 200 rpm.

**Flow cytometry (Bacterial cell count analysis)**

Count the number of events in the bead region to accurately estimate the volume analyzed in the data file. The microsphere standard contains approximately 1.0 × 10^8^ beads/mL; thus, after ~100-fold dilution into the stained cell preparation, the microsphere density is 1.0 × 10^6^ beads/mL, and one bead represents 10^–6^ mL. The number of signals in the bacteria region divided by the number of signals in the bead region provides the total number of bacteria per 10^–6^ mL in the flow cytometry analysis tube. Likewise, the number of events in the live bacteria region (or dead bacteria region) divided by the number of events in the bead region provides the number of live bacteria (or dead bacteria) per 10^–6^ mL in the flow cytometry analysis tube.

To calculate the density in the bacterial culture, the concentration in the flow cytometry tube must be multiplied by the dilution factors, including the 100-fold dilution at previous analysis steps and any further dilutions made due to a turbid culture.

$$bacteria/mL=\frac{(\left( \# of events in bacteria region \right) \times(dilution factors)}{((\# of events in bead region )\times10-6)}$$

**Transcriptomics study.** Log_2_fold-change values for global gene expression through RNAseq are presented in Table S1. Differential expressions of 14 target genes in *E. coli* O157:H7 were studied relative to expression of three housekeeping genes are presented in Fig. 8 of the main manuscript. Functions of all the genes studied and primers sequences used for this study through q-PCR are listed in Table S2.

**Table S1** The relative expression of a few important genes involved in quorum sensing, pathogenicity, and growth of *E. coli* 0157:H7. The significant (p<0.05) up and down regulation of genes were denoted as *.

| **Genes** | **Growth condition** | **Treatment** | **Log FC** | **Adjusted P value** | **Function** |
| --- | --- | --- | --- | --- | --- |
| *lsrA* | Biofilm | BF_NP(-)  BF_NP(+) | 0.0539  0.9155^*^ | 0.9719  0.0125 | Genes of lsr operon are associated with transportation of autoinducer II (AI-2) inside the cell for downstream quorum sensing process and pathogenicity |
| *lsrB* |  | BF_NP(-)  BF_NP(+) | 0.6426  1.0721^*^ | 0.2211  0.0006 |  |
| *lsrC* |  | BF_NP(-)  BF_NP(+) | 0.4829  1.0434^*^ | 0.5471  0.0029 |  |
| *lsrD* |  | BF_NP(-)  BF_NP(+) | 0.6151  0.9440^*^ | 0.3518  0.0094 |  |
| *lsrF* |  | BF_NP(-)  BF_NP(+) | 0.6530  0.9317^*^ | 0.2100  0.0041 |  |
| *lsrG* |  | BF_NP(-)  BF_NP(+) | 0.4082  0.6275^*^ | 0.4486  0.0267 |  |
| *lsrK* |  | BF_NP(-)  BF_NP(+) | 0.5095  0.8414^*^ | 0.3108  0.0025 |  |
| *eutC* | Biofilm | BF_NP(-)  BF_NP(+) | 4.5115^*^  -0.2092 | 1.42×E^27^  0.8011 | Eut operon genes involved in Ethanolamine (EA) metabolism which confer advantage during colonization on the gastrointestinal surface |
| *eutB* |  | BF_NP(-)  BF_NP(+) | 4.5826^*^  -0.2787 | 2.24×E^26^  0.7295 |  |
| *eutL* |  | BF_NP(-)  BF_NP(+) | 4.4275^*^  -0.0838 | 2.43×E^26^  0.9339 |  |
| *eutM* |  | BF_NP(-)  BF_NP(+) | 4.1518^*^  0.4683 | 7.40×E^26^  0.4613 |  |
| *eutP* |  | BF_NP(-)  BF_NP(+) | 4.2482^*^  0.9179 | 9.64×E^25^  0.1047 |  |
| *eutN* |  | BF_NP(-)  BF_NP(+) | 5.4583^*^  -0.1202 | 7.32×E^23^  0.9308 |  |
| *eutQ* |  | BF_NP(-)  BF_NP(+) | 4.4723^*^  0.7954 | 7.56×E^23^  0.2282 |  |
| *flgA* | Biofilm | BF_NP(-)  BF_NP(+) | -0.5722  -0.9972^*^ | 0.2975  0.0011 | Flagellar assembly and activity related genes involve in bacterial motility and biofilm formation |
| *flgB* |  | BF_NP(-)  BF_NP(+) | -0.8527  -1.4717^*^ | 0.2834  0.0010 |  |
| *flgC* |  | BF_NP(-)  BF_NP(+) | -0.7507  -1.4471^*^ | 0.5792  0.0196 |  |
| *motB* |  | BF_NP(-)  BF_NP(+) | -0.9118^*^  -1.6218^*^ | 0.0255  3.42×E^8^ |  |
| *motA* |  | BF_NP(-)  BF_NP(+) | -0.8686^*^  -1.6372^*^ | 0.03332  1.68×E^8^ |  |
| *fliA* |  | BF_NP(-)  BF_NP(+) | -0.9899^*^  -2.0427^*^ | 0.0986  7.93×E^8^ |  |
| *ftsL* | Planktonic | PL_NP(-)  PL_NP(+)  PL_NP(0) | -0.5073^*^  -0.12493  0.710553^*^ | 0.0297  0.7165  0.0001 | Genes involved in growth and cell division |
| *dksA* |  | PL_NP(-)  PL_NP(+)  PL_NP(0) | -0.42893  -0.1585  0.534819^*^ | 0.1015  0.6591  0.0099 |  |
| *ftsZ* |  | PL_NP(-)  PL_NP(+)  PL_NP(0) | 0.028942  0.131378  1.275575^*^ | 0.9347  0.642  7.73×E^16^ |  |
| *phnI* | Planktonic | PL_NP(-)  PL_NP(+)  PL_NP(0) | 2.2701^*^  2.4443^*^  -3.1543^*^ | 0.0001  2.98×E^5^  8.74×E^7^ | Phosphonate operon genes involved in organophosphonate utilization |
| *phnE* |  | PL_NP(-)  PL_NP(+)  PL_NP(0) | 2.1521  2.2886  -4.0479^*^ | 0.0854  0.0619  0.0002 |  |
| *phnM* |  | PL_NP(-)  PL_NP(+)  PL_NP(0) | 2.1114^*^  2.2673^*^  -1.8148^*^ | 0.0004  0.0001  0.0016 |  |
| *phnF* |  | PL_NP(-)  PL_NP(+)  PL_NP(0) | 2.4153^*^  2.6791^*^-3.9100^*^ | 4.20×E^6^  1.72×E^7^  1.58×E^6^ |  |
| *phnL* |  | PL_NP(-)  PL_NP(+)  PL_NP(0) | 2.4026^*^  2.7220^*^  -3.5176^*^ | 0.0002  2.24×E^5^  3.26×E^5^ |  |
| *phnK* |  | PL_NP(-)  PL_NP(+)  PL_NP(0) | 2.4399^*^  2.5568^*^  -3.0732^*^ | 0.0002  0.0002  0.0001 |  |
| *phnP* |  | PL_NP(-)  PL_NP(+)  PL_NP(0) | 1.0068^*^  1.0063^*^  -2.3247^*^ | 0.0244  0.0234  5.03×E^8^ |  |

**Table S2** Primer sequence and gene functions of selected target genes in *E. coli*, amplified for relative gene expression studies.

| **Genes** | **Primer sequence** | **Melting temperature** | **Gene function** |
| --- | --- | --- | --- |
| **Housekeeping genes** | | | |
| *16srRNA* | F: 5'-GGCTGAAAAGCTGCATTACC  R: 5'-CATCAGGCCGATGTTACCTT | 63.6  63.8 | 16S ribosomal RNA, component of 30S subunit of bacterial ribosome. |
| *gapA* | F: 5'-GCTTCCCAGAACATCATCCC  R: 5'-ACGGTCAGGTCAACTACGG | 65.7  62.7 | Codes for D-glyceraldehyde-3-phosphate dehydrogenase. |
| *mdh* | F: 5'-CCCGTTTCTTCTCTCAGCC  R: 5'-GCGTATCCAGCATACCTTCC | 63.9  63.3 | Codes for malate dehydrogenase, inversely proportional to the cell growth rate. |
| **Virulence and adhesion-factor genes** | | | |
| *stx1A* | F: 5'-CCATTCTGGCAACTCGCG  R: 5'-GGCAAGAGCGATGTTACGGT | 68.1  66.2 | Codes for Shiga toxin 1A subunit. |
| *stx2A* | F: 5'-TTGCTGTGGATATACGAGGGC  R: 5'-TCCGTTGTCATGGAAACCG | 65.9  67 | Codes for Shiga toxin 2A subunit. |
| *eaeA* | F: 5'-ATGCTTAGTCGTGGTTTAGG  R: 5'-GCCTTCATCATTGCGCTTTC | 58.5  63.5 | Outer membrane adhesion protein intimin, needed for attachment to host cells. |
| **Stress-response genes** | | | |
| *rpoS* | F: 5'-GATGACGTCAGCCGTATGCTT  R: 5'-GAGGCCAATTTCACGACCTAC | 66.3  64.5 | General stress regulator encoding σ – S, regulates several genes/operons.  Provides resistance to stationary phase cells. |
| *oxyR* | F: 5'-GAAGCACAGACCCACCAGTT  R: 5'-CAAACAACGGCACTTCAATG | 64.2  64.1 | Regulon of hydrogen peroxide-inducible genes. |
| **Biofilm formation genes** | | | |
| *flhD* | F: 5'-ATCGTCTGGTGGCTGTCAA  R: 5'-GTCCGCTATGTTTCGTCTCG | 64.5  64.6 | Transcriptional activator of flagellar genes, required for transcription of the other important class II operons. |
| *fliA* | F: 5'-GCTGGCTGTTATTGGTGTCG  R: 5'-CAACTGGAGCAGGAACTTGG | 65.5  64.8 | Regulates flagellar operons positively. |
| *motA* | F: 5'-CTTCCTCGGTTGTCGTCTGT  R: 5'-CTATCGCCGTTGSGTTTGGT | 64.3  63.9 | Stator element of the flagellar motor complex required for rotation of the flagellar motor (transmembrane proton channel) |
| *fimH* | F: 5'-GCGATGATTTCCAGTTTGTG | 63.1 | Codes for a mannose-binding adhesin present at the tip of type 1 pili, that allows binding to epithelial glycoproteins. |
|  | R: 5'-ATTGGCACTGAACCAGGGTA | 64.3 |  |
| *luxS* | 5'-ACCGTGTTCGATCTGCGCTT  5'-GCAAACAGGTGCTCCAGGGT | 69.2  68.7 | Codes for autoinducer-II, mediates quorum sensing. |
| *Crl* | 5'-GCGTGAGTTTCACGAGAAGCT  5'-TCATCCGCCGGTTCCA | 65.8  68.0 | Stimulates the transcription of csgBA, the operon encoding for the two curli subunits, in a sigma- factor dependent manner. |
| *fliC* | F: 5'-TTCGCAGCATCACTGGATTC  R: 5'-CATCGCAAAAGCAACTCCTG | 66.3  65.7 | Codes for σ - regulators involved in the coordination of flagellar and type 1 pili synthesis, that helps in adhesion and invasion. |
| *bolA* | F: 5'-CCGTATTCCTCGAAGTAGTGG  R: 5'-GCAACCCTTCCCACTCCTTAA | 62.4  66.1 | Structural gene encoding transcription factor involved in the morphogenetic pathways, induced during transition to stationary phase of growth. |
| *chpB* | F: 5'-TGTTGTCCTGAGTCCGTTCA  R: 5'-CGTTCCTTTCTTCGTTGCTC | 64.5  63.8 | Encodes two genes: growth inhibitor and a suppressor for the killing action. (toxin-antitoxin system) |
